# Quasispecies Analyses of the HIV-1 Near-full-length Genome With Illumina MiSeq

**DOI:** 10.3389/fmicb.2015.01258

**Published:** 2015-11-12

**Authors:** Hirotaka Ode, Masakazu Matsuda, Kazuhiro Matsuoka, Atsuko Hachiya, Junko Hattori, Yumiko Kito, Yoshiyuki Yokomaku, Yasumasa Iwatani, Wataru Sugiura

**Affiliations:** ^1^Department of Infectious Diseases and Immunology, Clinical Research Center, National Hospital Organization Nagoya Medical CenterNagoya, Japan; ^2^Department of AIDS Research, Graduate School of Medicine, Nagoya UniversityNagoya, Japan

**Keywords:** HIV-1, deep sequencing, drug resistance, error correction, consensus sequence estimation, quasispecies

## Abstract

Human immunodeficiency virus type-1 (HIV-1) exhibits high between-host genetic diversity and within-host heterogeneity, recognized as quasispecies. Because HIV-1 quasispecies fluctuate in terms of multiple factors, such as antiretroviral exposure and host immunity, analyzing the HIV-1 genome is critical for selecting effective antiretroviral therapy and understanding within-host viral coevolution mechanisms. Here, to obtain HIV-1 genome sequence information that includes minority variants, we sought to develop a method for evaluating quasispecies throughout the HIV-1 near-full-length genome using the Illumina MiSeq benchtop deep sequencer. To ensure the reliability of minority mutation detection, we applied an analysis method of sequence read mapping onto a consensus sequence derived from *de novo* assembly followed by iterative mapping and subsequent unique error correction. Deep sequencing analyses of aHIV-1 clone showed that the analysis method reduced erroneous base prevalence below 1% in each sequence position and discarded only < 1% of all collected nucleotides, maximizing the usage of the collected genome sequences. Further, we designed primer sets to amplify the HIV-1 near-full-length genome from clinical plasma samples. Deep sequencing of 92 samples in combination with the primer sets and our analysis method provided sufficient coverage to identify >1%-frequency sequences throughout the genome. When we evaluated sequences of *pol* genes from 18 treatment-naïve patients' samples, the deep sequencing results were in agreement with Sanger sequencing and identified numerous additional minority mutations. The results suggest that our deep sequencing method would be suitable for identifying within-host viral population dynamics throughout the genome.

## Introduction

Knowledge of the genome sequence of human immunodeficiency virus type-1 (HIV-1) is fundamental for improving the clinical outcome of patients infected with HIV-1 and for understanding viral co-evolution within hosts. However, not only between-host HIV-1 genetic diversity and within-host viral heterogeneous population make it difficult to determine the viral sequences within host. HIV-1 is classified into four groups (M, N, O, and P), and the group that is most widespread globally, M, is further divided into nine subtypes (A, B, C, D, F, G, H, J, and K), with more than 70 circulating recombinant forms (CRFs), according to the Los Alamos HIV Sequence database (http://www.hiv.lanl.gov/), and numerous unique recombinant forms (URFs; Sharp, 2002; Taylor et al., 2008; Hemelaar et al., 2011; Sharp and Hahn, 2011). Genetic diversity between the subtypes ranges from 25 to 35% (Korber et al., 2001), which is extremely high compared to the human population, in which < 1% of distinct DNA sequences are distinct (International HapMap, 2003, 2004). This diversity is considered a consequence of HIV-1's short replication period, lack of proofreading machinery, and recombination in viral replication (Robertson et al., 1995; Perelson et al., 1996; Blackard et al., 2002). The genetic diversity of HIV-1 likely influences the effectiveness of antiretroviral therapy (Wainberg and Brenner, 2012) and at least partially prevents the development of curable strategy against HIV-1 infection (Thomson et al., 2002). Moreover, the error-prone replication induces a within-host genetically diverse heterogeneous viral population, recognized as quasispecies (Ojosnegros et al., 2011). The quasispecies are considered a source of drug-resistant or immune escape variants. Within-host minority viruses likely influence clinical outcome (Johnson et al., 2008; Balduin et al., 2009; Geretti et al., 2009; Metzner et al., 2009; Simen et al., 2009; Paredes et al., 2010), although some reports have found no association between treatment failure and minority variants (Peuchant et al., 2008; Jakobsen et al., 2010; Metzner et al., 2010; Stekler et al., 2011).

To improve clinical outcomes and further understand viral co-evolution within-hosts, the HIV-1 RNA genome has been sequenced using the direct Sanger sequencing method. For example, before treatment against HIV-1 infection, the *pol* and *env* V3 regions are sequenced in genotyping resistance tests and tropism tests that predict viral susceptibility to antiretroviral drugs (Smit, 2014). However, analysis of viral polymorphic sequences is limited using Sanger sequencing method. For example, direct Sanger sequencing is inappropriate for analyzing regions containing heterogeneous insertions or deletions, such as *gag* and *env*. Within-host quasispecies population analyses using direct Sanger sequencing can detect low-frequency mutations in only up to 10–20% of the population. In addition, primer design may be troublesome when analyzing sequences of large or multiple segments.

Recently developed next-generation sequencing technologies that output unprecedented amounts of short sequence reads enable the analysis of viral quasispecies in further depth (Willerth et al., 2010; Dudley et al., 2012; Gall et al., 2012; Henn et al., 2012; Gibson et al., 2014; Park et al., 2014). Bench-top deep sequencers Roche/454 GS Junior and Ion PGM, both based on the pyrosequencing method, are applicable for analyses of limited regions of the HIV-1 genome (Dudley et al., 2012; Gibson et al., 2014; Park et al., 2014). Illumina has released another bench-top deep sequencer, MiSeq, based on bridge sequencing technology, which, compared with the aforementioned pyrosequencing platforms, can output large amounts of sequence reads with a lower intrinsic error rate, especially at homopolymeric regions, including the drug-resistance-related reverse transcriptase (RT) K65 codon (Varghese et al., 2010; Loman et al., 2012; Junemann et al., 2013). Here, we have proposed a practical method to analyze viral quasispecies of the HIV-1 near-full-length genome in clinical samples using the Illumina MiSeq deep sequencing method and especially evaluated nucleotide variations in viral sequences of the Pol and the Env V3 encoding regions.

## Materials and methods

### Plasmid sample preparation

To examine analytical biases that may produce misleading results and intrinsic errors in sequence reads from Illumina MiSeq, pNL4-3 (pNL4-3_wt_) was used as a reference clone. Furthermore, to examine the threshold of deep sequencing in detecting minority mutations in clinical samples, artificially simulated samples were prepared by mixing multiple different clones. For this purpose, three pNL101-based recombinant infectious clones (Neuveut and Jeang, 1996) possessing drug-resistance mutations RT K103N (pNL101_rtK103N_), RT M184V (pNL101_rtM184V_), and integrase (IN) Q148H (pNL101_inQ148H_) were constructed using standard site-directed mutagenesis protocols as described previously (Hachiya et al., 2008; Shimura et al., 2008). Seven ratios of pNL4-3_wt_: pNL101_rtK103N_: pNL101_rtM184V_: pNL101_inQ148H_ were mixed as follows: (a) 100:0:0:0, (b) 99.97:0.01:0.01:0.01, (c) 99.7:0.1:0.1:0.1, (d) 98.5:0.5:0.5:0.5, (e) 97:1:1:1, (f) 70:10:10:10, and (g) 40:20:20:20.

### Clinical sample collection and Sanger sequencing

Fifty-two plasma samples were collected from 33 HIV-1-infected patients who visited the Nagoya Medical Center in Japan from January 2009 to April 2014 (Supplementary Table S1). Forty-five plasma samples collected from 25 patients enrolled in the Japanese Drug Resistance HIV-1 Surveillance Network (Gatanaga et al., 2007; Hattori et al., 2010; Shiino et al., 2014) were also used in this study. Thus, a total of 97 plasma samples obtained from 58 patients were used. To monitor viral quasispecies chronologically, plasma samples were obtained at 10 time points from one patient failing protease inhibitors (PIs) containing regimens. The total RNA was extracted from 200- or 400-μL of the plasma sample using the MagNA Pure Compact Nucleic Acid Isolation Kit I (Roche Diagnostics K.K., Tokyo, Japan). Extracted RNA was eluted in a final volume of 50 μL of elution buffer and used for subsequent analyses. HIV-1 protease (PR) (297 bps; 2253–2549, positions based on HXB2 numbering), RT (720 bps; 2550–3269) and *env* V3 (108 bps; 7110–7217) sequences of each sample were analyzed using the bulk sequencing method as previously reported (Gatanaga et al., 2007; Hattori et al., 2010; Shiino et al., 2014). Drug-resistance mutations in Pol were determined according to the list reported by Shafer et al. (Bennett et al., 2009) and IAS-USA (Wensing et al., 2014). HIV-1 subtypes were determined using phylogenetic analysis with reference sequences recommended by the Los Alamos Database (http://www.hiv.lanl.gov/). Genotypic tropism tests were performed using geno2pheno [coreceptor] (http://coreceptor.geno2pheno.org/) with a false-positive rate of 10%.

This study was conducted according to principles in the Declaration of Helsinki. The Ethical Committee at the National Institute of Infectious Diseases and Nagoya Medical Center in Japan approved the study. All patients provided written informed consent for the collection of samples and subsequent analyses.

### Amplification of the HIV-1 near-full-length genome in clinical samples

**Figure 1:**
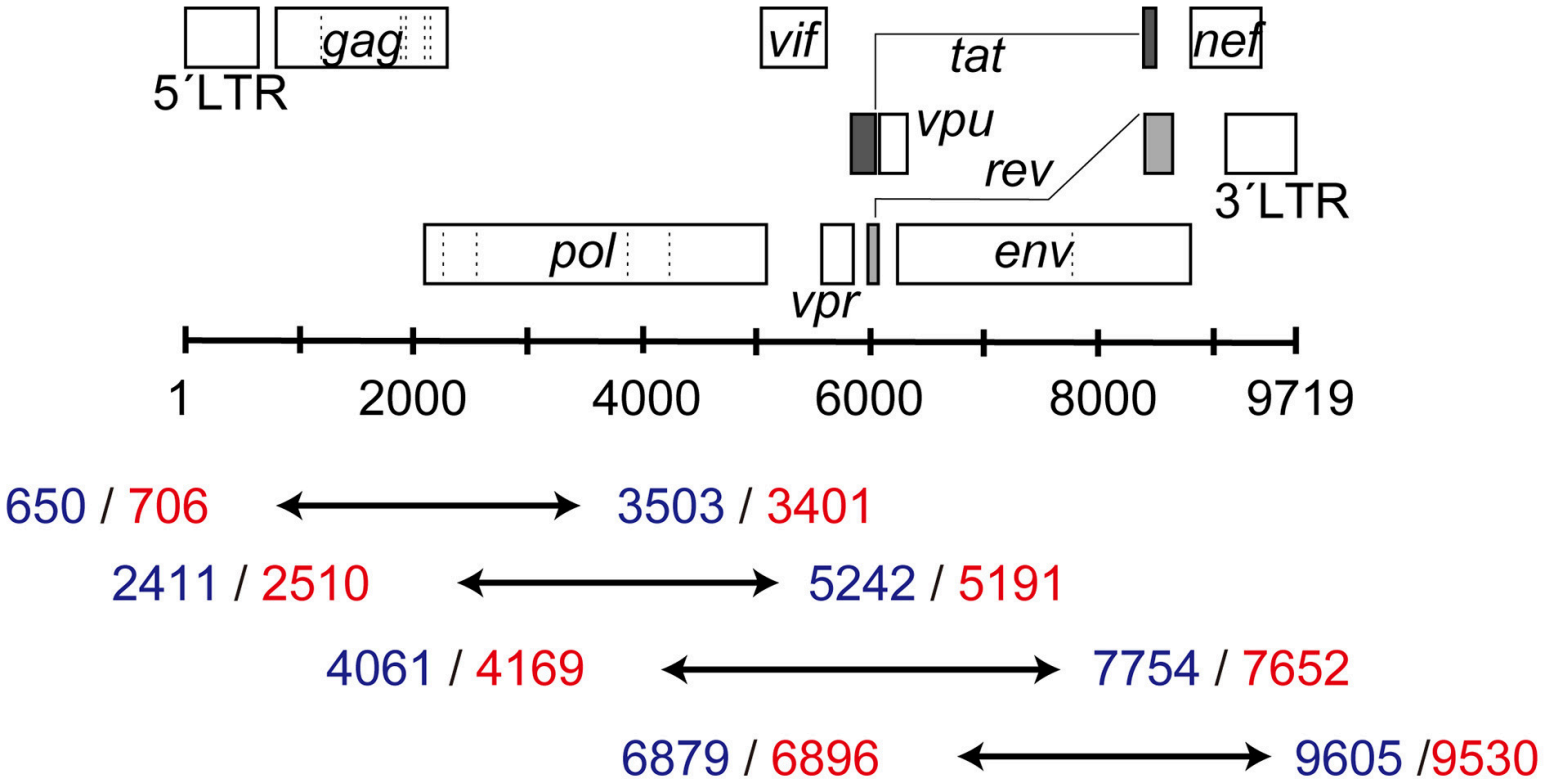
**Four overlapping segments covering the HIV-1 near-full-length genome**. The blue numbers represent the 5′- and 3′-end positions (HXB2 numbering) for RT-PCR, whereas red numbers show those for nested PCR.

To analyze the full-length HIV-1 genome (excluding LTR regions) using MiSeq, the *gag* to *nef* (8825 bps; 706–9530) region of the genome was amplified in four overlapping segments, as shown in Figure 1. The primer sequences used for the amplifications are listed in Supplementary Table S2. RT-PCR was performed using a PrimeScript II High Fidelity One Step RT-PCR Kit (Takara, Shiga, Japan), followed by nested PCR using PrimeSTAR GXL DNA Polymerase (Takara, Shiga, Japan). Finally, four amplified PCR products were combined into one sample with a MultiScreen HTS PCR96 filter plate according to the manufacturer's instructions (Merck Millipore, Billerica, Massachusetts, USA). The purified DNA was eluted into a final volume of 50 μL of distilled water.

### Library preparation and deep sequencing with illumina MiSeq

Viral DNA libraries for deep sequencing were prepared using the Nextera DNA Sample Prep Kit (Illumina K.K., Tokyo, Japan) according to the manufacturer's instructions. Unamplified DNA of the recombinant clones and amplified DNA obtained from clinical samples were used for the preparation. The prepared library was sequenced using Illumina MiSeq, generating paired-end 2 × 250-bps-long sequence reads. For each run, a maximum of 24 samples were concurrently examined.

### Sequence read analysis for deep sequencing

The sequence reads obtained from Illumina MiSeq were analyzed following three procedures: (I) mapping of the sequence reads onto a reference sequence or a consensus sequence; (II) error correction; and (III) frequency estimation of bases, amino acids, or short (< 250-bps-long) fragment sequences. The detailed methods at each step are described below.

- Consensus Sequence Estimation and Read Mapping. Sequence read mapping was conducted with the BWA-MEM algorithm implemented in the BWA-0.6.4 program (Li and Durbin, 2009, 2010) using the default setting. We performed four sequence read mapping methods (Supplementary Figure S1A) as follows:
  - Simple mapping onto an infectious clone sequence. The sequence reads were mapped on a given infectious clone sequence, such as NL4-3, HXB2, JRCSF (GenBank accession no. M38429) in subtype B, and 93JP-NH1(NH1) in CRF01_AE (AB052995).
  - Mapping onto consensus sequence derived from *de novo* assembly. Long fragments (>1000 bps) of the HIV-1 near-full-length genome sequence were assembled *de novo* from sequence reads with the VICUNA program (Yang et al., 2012; Malboeuf et al., 2013). The estimated long fragment sequences were mapped onto the HXB2 sequence with the BWA program. Next, the long fragment sequences were connected to construct a near-full-length consensus sequence. Sequence reads were then mapped onto the constructed consensus sequence.
  - Mapping onto consensus sequence estimated from iterative mapping (McElroy et al., 2014; Verbist et al., 2015). The first cycle of iterative mapping was performed on a given reference sequence, such as NL4-3, HXB2, JRCSF, or NH1, with BWA using an option of “-B 1” to reduce mismatch penalty for mapping. The majority base at each position was accepted as the consensus base. In cases of insertions or deletions, the longer sequences were always chosen for consensus sequence estimation; i.e., deletions were ignored and insertions were accepted regardless of their prevalence. The consensus sequence estimated in the first cycle was used as a reference sequence of the second cycle of iterative mapping with the BWA program at the default setting. This procedure was repeated nine times to refine a consensus sequence, and, finally, the sequence reads were mapped onto the consensus sequence obtained in the 9th iterative mapping.
  - Combination of (ii) and (iii): Sequence reads were mapped onto the consensus sequence estimated from *de novo* assembly using the VICUNA program (Yang et al., 2012; Malboeuf et al., 2013) followed by the iterative mapping (McElroy et al., 2014; Verbist et al., 2015).
- Error Correction. We performed error correction using averaged quality score (QS)-values for each reference sequence position. The detailed method is described below.
- Frequency Estimation. We estimated the frequency of each base at a given position by counting the number of nucleotides for each base that remained after error correction. We also calculated the occupancy of sequences within a < 250-bps-long range because the maximum length of Illumina MiSeq sequence reads is 250 bp. First, we extracted short fragment sequences from the mapped sequence reads within the targeted range. Then, identical fragment sequences were grouped into a haplotype sequence. The number of the fragment sequences was counted for each haplotype sequence. Next, haplotype sequences including bases of averaged QSs below 20 were removed. The remaining haplotype sequences were used for frequency estimation.

## Results

### Use of the consensus sequence estimated from *de novo* assembly or iterative mapping improved the sequence read mapping results

**Figure 2:**
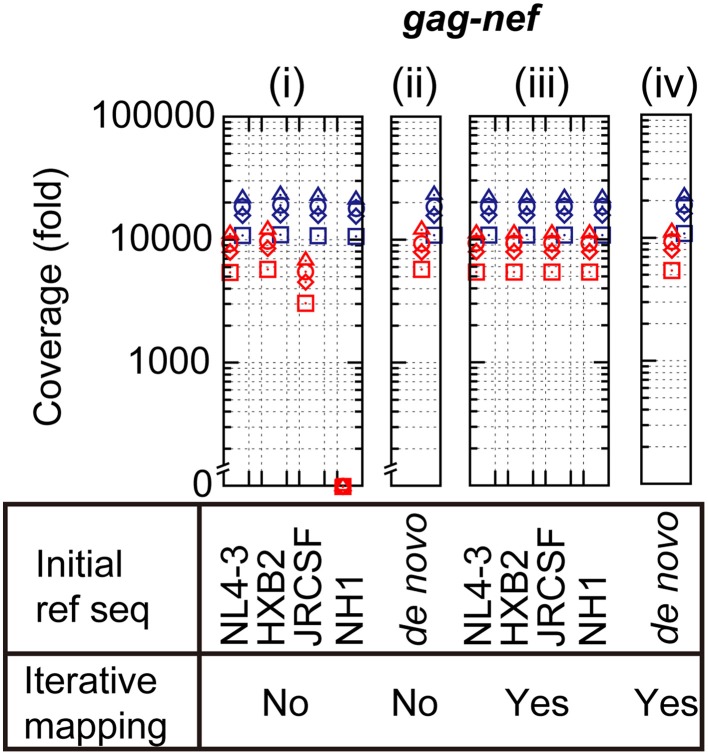
**Analytical biases at read mapping**. The red and blue symbols represent minimum and maximum coverage in the *gag-nef* region. The sequence read coverage from four deep-sequencing runs is plotted using squares, triangles, rhombuses, and circles.

To examine analytical bias that may produce misleading results and an intrinsic error rate of output sequence reads from Illumina MiSeq, pNL4-3_wt_ was deeply sequenced in quadruplicate. The obtained sequence reads from each of the quadruplicate runs were individually mapped onto the original pNL4-3_wt_ sequence [Schema (i) in Supplementary Figure S1A]. The mapping results demonstrated >5000-fold coverage throughout the HIV-1 genome (Figure 2, Supplementary Figure S1B and Supplementary Table S3). To investigate the effect of selected reference sequences on the mapping accuracy and coverage of the sequence reads, two different subtype B sequences, HXB2 (97.4% identical to NL4-3) and JRCSF (93.8%), and one CRF01_AE sequence, NH1 (85.0%), were selected as reference sequences for mapping [Schema (i) in Supplementary Figure S1A]. As shown in Figure 2, the mapping coverage by HXB2 (5722- to 23,058-fold) and JRCSF(3038- to 22,570-fold) were comparable to that of NL4-3 (5424- to 21,616-fold), whereas a considerable reduction in coverage was observed when NH1 was used as the reference sequence (0- to 21,064-fold). Thus, selection of the reference sequence is clearly a critical factor for accurate mapping and high coverage of deep sequencing reads. However, for clinical samples, selection of an appropriate reference sequence is problematic because the sample sequences are unknown at the time of deep sequencing. To overcome this problem, we estimated a consensus sequence from *de novo* assembly, iterative mapping, or *de novo* assembly followed by iterative mapping (Yang et al., 2012; Malboeuf et al., 2013; Gibson et al., 2014; McElroy et al., 2014; Verbist et al., 2015) [Schema (ii)–(iv) in Supplementary Figure S1A]. We used the resulting consensus sequence as the reference sequence for mapping, as previously proposed by others (Yang et al., 2012; Malboeuf et al., 2013; Gibson et al., 2014; McElroy et al., 2014; Verbist et al., 2015). The use of the *de novo* assembled consensus sequence provided comparable mapping results (5731- to 23,053-fold) to that of NL4-3. The assembled consensus sequence was analogous to the original NL4-3 reference sequence but included ~10 *nef* mutations, suggesting that *de novo* assembly is likely insufficient to estimate the true consensus sequence. By contrast, iterative mapping or *de novo* assembly followed by iterative mapping estimated a consensus sequence that was identical to the NL4-3 sequence and resulted in the same mapping coverage as that achieved using NL4-3 as the reference sequence. These results suggest that iterative mapping and *de novo* assembly followed by iterative mapping can estimate the true consensus sequence and are most appropriate for sequence read mapping. Hence, in the following sections, we mapped sequence reads from deep sequencing using consensus sequence estimation by *de novo* assembly followed by iterative mapping (Yang et al., 2012; Malboeuf et al., 2013; Gibson et al., 2014; McElroy et al., 2014; Verbist et al., 2015).

### Our unique error-correction method reduced the prevalence of erroneous bases found in sequence reads for a recombinant clone below 1% in each sequence position

**Figure 3:**
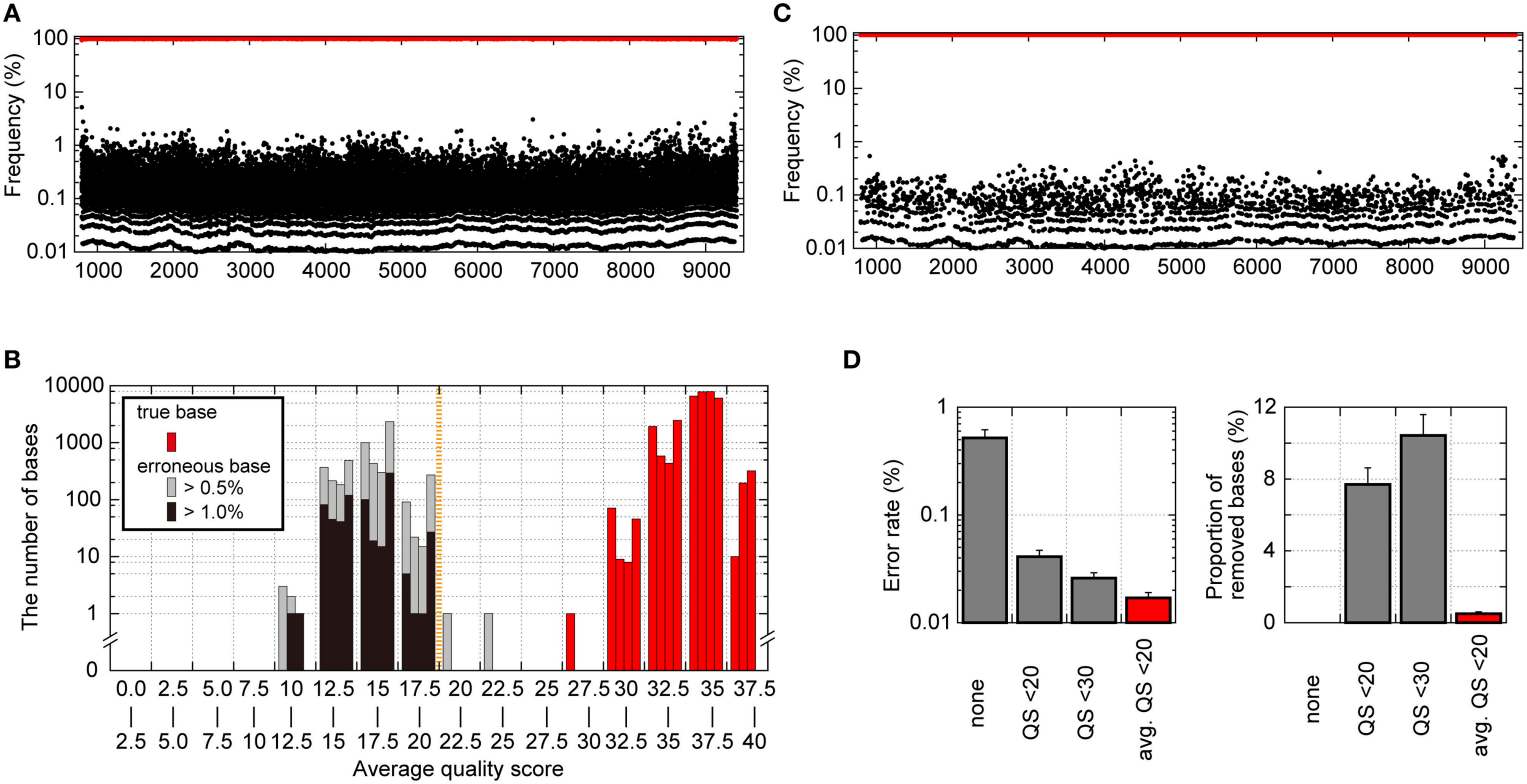
**Analytical bias in pNL4-3_wt_ sequence reads from the Illumina MiSeq deep sequencer**. **(A)** Frequencies of nucleotides with true bases (red dots) and with erroneous bases (black dots) throughout the pNL4-3_wt_ genome except for the LTR regions prior to any error correction. **(B)** Histogram of averaged QSs for true bases and erroneous bases at each genome position. The averaged QSs for true bases are shown in red bars, whereas those for erroneous bases occupying >0.5 and >1.0% of the population are represented as gray and black bars, respectively. In each range (x-axis) of the histogram, the four bars representing results from each quadruplicate runs are shown. **(C)** Frequencies of nucleotides with true bases (red dots) and erroneous bases (black dots) throughout the pNL4-3_wt_ genome after error correction removed each base with averaged QSs below 20 at the respective position. **(D)** Error rate after error correction and the proportion of removed nucleotides among all nucleotides using error correction. Results are shown for no error correction, quality-filtering error correction by removing nucleotides with QSs below 20 or 30, and our error correction removing bases of averaged QSs below 20.

We also analyzed intrinsic errors in the sequence reads from deep sequencing for pNL4-3_wt_. As shown in Figure 3A, without any error-correction handling, even with clonal pNL4-3_wt_ sequencing results, the erroneous bases occupied a maximum of 6.4% of the population at each reference sequence position. The erroneous bases induced drug-resistance-associated mutations such as IN T66A/I/K and Q148H/K/R at maximums of 1.7 and 1.6% of the population. Considering minority mutation detection by deep sequencing, this error rate is excessive, making minority-mutation determinations inaccurate. In analyzing the patterns of errors, a dominant pattern was substitution (~99.6% in total errors), whereas insertions or deletions were not frequently observed, as previously reported by others (Loman et al., 2012; Junemann et al., 2013). Further examination of substitution patterns revealed that C>A and T>G transversion errors were most frequently observed in the pNL4-3_wt_ sequence reads (Supplementary Figure S2). The predominance of T>G transversion was previously reported for MiSeq (Schirmer et al., 2015) and Illumina Genome Analyzer (Nakamura et al., 2011; Flaherty et al., 2012), both of which are based on the bridge sequencing method, suggesting that the transversion errors might be intrinsic to the apparatuses and the technology.

To improve the accuracy of the sequencing results, we sought to establish a novel error correction method by distinguishing true minority bases from erroneous bases. To differentiate true and erroneous bases, we focused on Phred QS-values of nucleotides in pNL4-3_wt_ sequence reads. QSs of 10, 20, and 30 indicate 90, 99, and 99.9% base-calling accuracy, respectively. Nucleotides with true bases tend to demonstrate high QS-values, whereas low QS-values are associated with erroneous bases (Supplementary Figure S3). When the QSs of nucleotides with true bases were averaged at the respective reference sequence positions, the averaged QS-values for true bases were >25, suggesting >99.7% base-calling accuracies (Figure 3B). By contrast, when we focused on bases occupying >1% of the population at the respective positions, the averaged QS-values for erroneous bases were below 20 and were clearly different from those for true bases at a threshold of 20 (Figure 3B). Thus, this result indicates that “an averaged QS-value of 20” is a reasonable cut-off threshold to distinguish true and erroneous bases. Figure 3C shows the results of pNL4-3_wt_ sequencing managed by our novel error-correction method that removed bases with averaged QS-values below 20 at each reference sequence position (Supplementary Figure S3), and the erroneous bases did not occupy >0.54% of the population. Especially, each population of the drug resistant mutations in Pol and erroneous sequences at Env V3 and PR cleavage sites in Gag, Pol, Nef (Shafer and Schapiro, 2008; Fun et al., 2012) was not exceeded 0.2%. The error rates were reduced to 0.017 ± 0.002 from 0.52 ± 0.098% of the raw sequence reads (Figure 3D). Of note, our error-correction method removed only < 1% of all nucleotides (Figure 3D), suggesting selective removal of erroneous bases.

As a comparative method, we applied the simple quality-filtering correction method reported by others (Dudley et al., 2012; Gall et al., 2012; Pessoa et al., 2014) to the same pNL4-3_wt_ sequence reads. The quality-filtering method simply discards any nucleotides with a QS below 20 or 30 as a cut-off value (Supplementary Figure S3). This alternative method was also successful in reducing error rates to 0.041 ± 0.006 and 0.026 ± 0.003% with a cut-off of QS < 20 and QS < 30, respectively (Figure 3D). However, the quality-filtering method discarded 7% and 11% of all nucleotides by the QS < 20 and QS < 30 cut-offs, respectively (Figure 3D).

### Deep-sequencing analyses coupled with our analysis method for distinct recombinant clone mixtures successfully detected minority mutations with a prevalence of >1%

**Figure 4:**
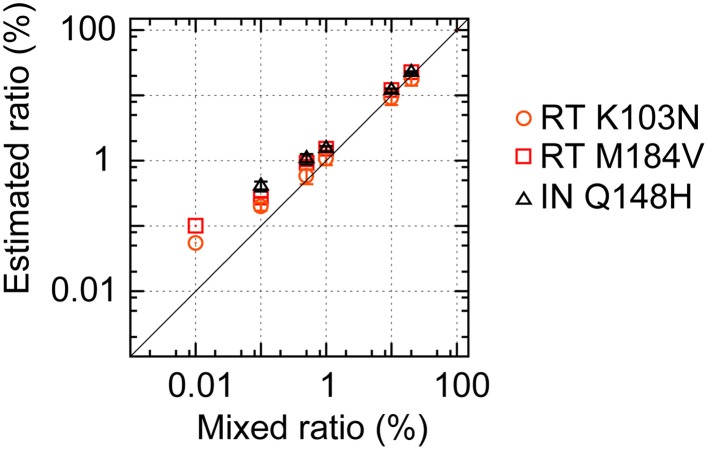
**Deep sequencing to detect minority viral populations**. pNL4-3_wt_ and pNL101-based mutant clones (pNL101_rtK103N_, pNL101_rtM184V_, and pNL101_inQ148H_) were mixed at seven different ratios: (a) 100:0:0:0, (b) 99.97:0.01:0.01:0.01, (c) 99.7:0.1:0.1:0.1, (d) 98.5:0.5:0.5:0.5, (e) 97:1:1:1, (f) 70:10:10:10, and (g) 40:20:20:20. The estimated frequencies of RT K103N, RT M184V, and IN Q148H are shown in orange circles, red squares, and black triangles, respectively.

To confirm the potential of our mapping and error-correction methods, we performed deep sequencings of mixtures of four recombinant clones, pNL4-3_wt_, pNL101_rtK103N_, pNL101_rtM184V_, and pNL101_inQ148H_ in triplicate, at seven different ratios: (a) 100:0:0:0, (b) 99.97:0.01:0.01:0.01, (c) 99.7:0.1:0.1:0.1, (d) 98.5:0.5:0.5:0.5, (e) 97:1:1:1, (f) 70:10:10:10, and (g) 40:20:20:20 (Figure 4, Supplementary Table S4). We successfully detected three amino acid mutations stably in mixture samples when mutant clones were mixed at ≥0.5% prevalence [(d)–(g)]. For samples (b) and (c), where mutant clones were mixed at 0.01 and 0.1% prevalence, one and two in triplicate tests identified the three mutations, respectively. During the amino acid population analyses, our error-correction removed only ~3% of all three-nucleotide sequences. By contrast, the simple quality-filtering correction method with a QS < 20 or QS < 30 cut-off, removed ~25 or 27% of all three-nucleotide sequences, although the quality filtering correction method also allowed us to stably detect three mutations in samples (d)–(g), where mutant clones were mixed at ≥0.5% prevalence (Supplementary Table S4). Taken together with the aforementioned results on error prevalence, our analysis method enables us to detect amino acid mutations at >1% of the population reproducibly and semi-quantitatively while maximizing usage of the sequence read data.

Hence, in the following sections, errors in sequence reads from deep sequencing were corrected with our error-correction method based on averaged QS-values at a threshold of 20. Furthermore, we used error-corrected >1%-frequency bases, amino acids, or short < 250-bps-long fragment sequences.

### A near-full-length genome amplification protocol was successfully constructed

We designed primer sets to amplify near-full-length viral RNA genomes in four overlapping segments (Figure 1). A total of 97 clinical plasma samples were examined. Phylogeny analyses of *pol* sequences derived from Sanger sequencing indicated that 58 plasma samples from 22 patients and 39 samples from 36 patients contained subtype B and non-subtype B viral RNAs. The results showed that all of the four segments were successfully amplified for the subtype B samples with >200 copies/mL (Supplementary Table S5). By contrast, two subtype B samples with 50 and 65.7 copies/mL were incompletely amplified with missing segments. The same primer sets were tested for the remaining 39 non-subtype B viruses, including 10 subtype C, 10 CRF01_AE, 9 subtype F, and 10 CRF02_AG HIV-1. We successfully amplified all four segments for subtype C, CRF01_AE, subtype F, and CRF02_AG viral genomes from samples with up to 432, 700, 2980, and 1600 copies/mL, respectively (Supplementary Table S5). However, we failed to amplify three subtype F samples below 1240 copies/mL, suggesting lower amplification efficacy for non-subtype B viral genomes than subtype B genomes. In particular, amplification of the *in-env v5* regions in non-subtype B genomes was relatively unsuccessful. This is likely due to more nucleotide mismatches between the primer sequences and non-subtype B viral genome sequences (Supplementary Table S6). Further adjustment of these primers, especially for the *in-env v5* region, is required to improve amplification for non-subtype B viral genomes and to effectively amplify HIV-1 genomes regardless of their subtypes. Consequently, all four segments were successfully amplified from 92 plasma samples.

Subsequently, we deeply sequenced 92 amplified samples. When we analyzed the obtained sequence reads, >95% of the sequence reads were mapped onto the estimated consensus sequence for each sample. Further, as shown in Supplementary Figure S4, >1000-fold coverage of sequence reads were obtained at each position throughout *gag*-*nef*, except for the 5′-end of *matrix* in six samples, *env* in 1 sample, and the 3′-end of *nef* in 1 sample (Supplementary Table S7). Therefore, each minority nucleotide mutation occupying >1% of the population was confirmed from at least 10 sequence reads. Taken together, the results highlight that amplification with our primer sets followed by deep sequencing enabled us to analyze low-frequency mutations with sufficient sequence read coverage.

### Our deep sequencing method detected more minority variants than the direct Sanger sequencing method

**Figure 5:**
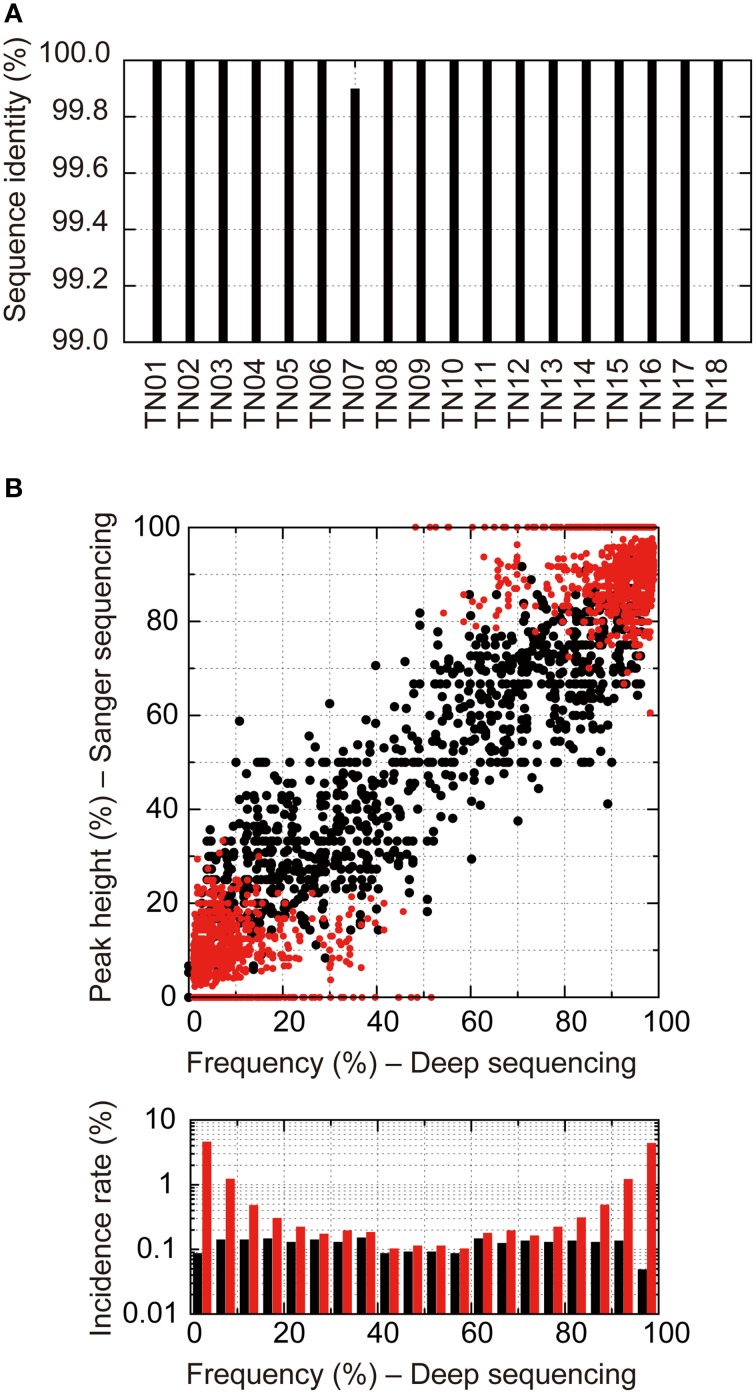
**Direct Sanger sequencing and deep sequencing comparison of 18 treatment-naïve samples**. **(A)** Identity of consensus nucleotide sequences at positions where A, T, G, or C was detected by Sanger sequencings between the two methods. **(B)** Correlation of occupancies of bases at the positions where multiple bases at >1% prevalence were detected by deep sequencings between the two methods. For Sanger sequencing, occupancies of bases were estimated from their electropherogram peak heights. Black dots show mixture bases assigned from Sanger sequencing electropherograms. Red dots show mixture bases that were unassigned by Sanger sequencing but were identified by the deep sequencing method. Bottom graphs represent histograms of occupancies detected by the deep sequencing method. The black and red bars show incident rates detected by Sanger sequencings and those detected only by deep sequencing methods, respectively.

To evaluate the potential of detecting quasispecies and minority population using our near-full-length deep sequencing method, we compared the results obtained with the proposed method and the direct Sanger sequencing method. We focused on sequences at PR-RT encoding regions (1017 bps; 2253–3269) from 18 treatment-naïve patients, including 13 subtype B, 4 CRF01_AE, and 1 CRF02_AG viruses (Supplementary Table S1). We achieved both deep and Sanger sequencings from the same amplicons. Only one mismatch was found in one sample (TN07) between the consensus sequences derived using the deep and Sanger sequencing methods (Figure 5A), except 216 positions in 18 samples where Sanger sequencing detected mixture bases. Thus, the concordance rate of the two methods was 99.994% [one mismatch in 18,090 (1017 × 18 – 216) positions]. Furthermore, we evaluated the sensitivity of the deep and Sanger sequencing methods in detecting minority populations. For Sanger sequencing, base occupancies were calculated from the electropherogram peak height ratios. As shown in Figure 5B, when base occupancies were analyzed at the 216 positions where Sanger sequencing detected mixture bases, a good correlation was observed between the Sanger sequencing method and deep sequencing method (1143 peaks, *R*^2^ = 0.76, *P* < 0.0001, single regression analysis; black dots in Figure 5B), and all mixture bases detected with Sanger sequencings were identified with the deep sequencing method. However, using the deep sequencing method, we detected an additional 1069 minority bases in all 18 samples that Sanger sequencing failed to recognize (red dots in Figure 5B). These results suggest that deep sequencing is more sensitive in detecting minority variants than Sanger sequencing and enables us to analyze quasispecies in clinical samples semi-quantitatively. These additional minority bases contained substitutions conferring drug resistance, and PR M46I (1.1% in TN03, 3.8% in TN04), RT T215S (1.0% in TN01), and RT K219R (1.2% in TN04) were identified. Thus, deep sequencing is likely useful in determining effective treatment regimen in clinical settings.

### Our deep sequencing method is applicable for detecting minority X4-tropic viruses and examining the chronological population dynamics of quasispecies

To confirm the clinical advantages of deep sequencing, we applied our deep sequencing method in genotypic tropism testing to determine the co-receptor usage of 18 treatment-naïve patients' samples (Supplementary Figure S5). The deep sequencing method identified heterogeneous V3 sequences in each sample (Supplementary Table S8) and identified nine samples possessing X4-tropic viruses, whereas only 5 samples were identified using Sanger sequencing. As shown in Supplementary Figure S5, all X4 tropic viral sequences in four samples diagnosed using the deep sequencing had less than a 20% minority population (Supplementary Table S8). Thus, the deep sequencing is more sensitive in detecting minority X4-tropic viruses than Sanger sequencings.

**Figure 6:**
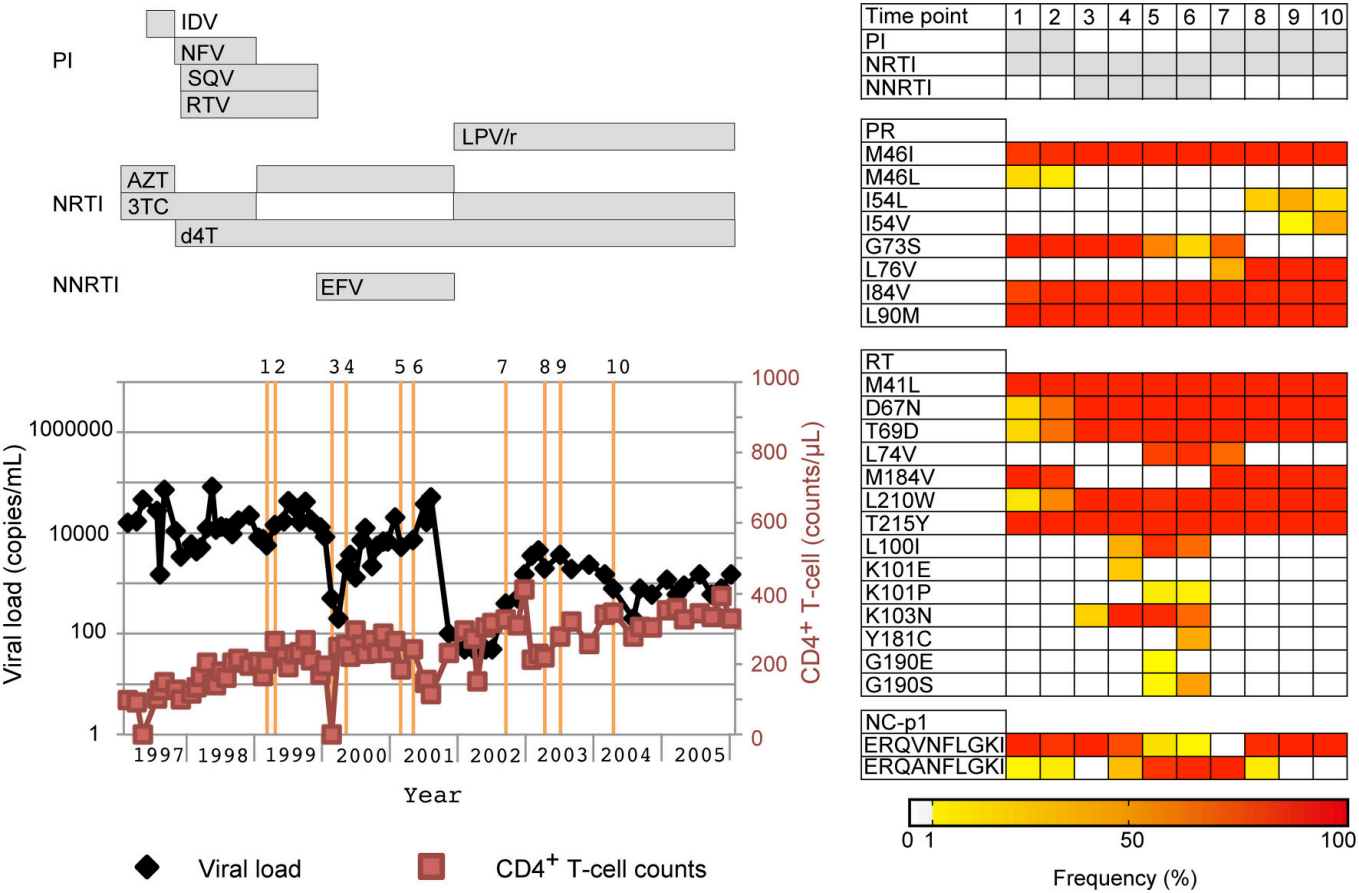
**Application of our deep sequencing method in examination of chronological population changes of drug-resistance mutations**. Chronological changes in viral loads, CD4^+^ T-cell counts, and occupancies of drug-resistance mutations for a patient who failed a PI-containing regime. In the left graph, viral loads and CD4^+^ T-cell counts are shown in black and light red lines, respectively. Collection dates of analyzed samples are highlighted in orange perpendicular lines. Over the orange line, the identification numbers for the collection dates are also shown. At the top of the graph, periods when drugs were used are shown in horizontal bars. Furthermore, population dynamics of resistance related mutations are shown on the right of the graph. The occupancies of the mutations are highlighted with colors according to the bottom bar. PI, PR inhibitor; NRTI, nucleoside/nucleotide RT inhibitor; NNRTI, non-nucleoside RT inhibitor. IDV, indinavir; NFV, nelfinavir; SQV, saquinavir; RTV, ritonavir; LPV/r, lopinavir boosted with ritonavir; AZT, azidothymidine; 3TC, lamivudine; d4T, stavudine; EFV, efavirenz.

To confirm whether our deep sequencing method allows us to dissect quasispecies population dynamics and identify minority drug-resistance mutations relevant to treatment failure, we retrospectively monitored changes in drug-resistance-related mutations in one multi-drug-resistant case (PI-resistant patient #1 in Supplementary Table S1) with M41L/D67N/T69D/M184V/L210W/T215Y in the RT region and M46IL/G73S/I84V/L90M in the PR region (Figure 6). We analyzed 10 time points and found that under an EFV-based regimen (time points 3–6), the prevalence of non-nucleoside RT inhibitor (NNRTI)-resistant mutations L100I and K103N increased from < 1 to 89.2% and 17.7 to 95.2%, respectively, with viral load relapse from 500 (time point 3) to 5400 copies/mL (time point 5). At time point 6 with 7200 copies/mL, the population of the other NNRTI-resistant mutations, Y181C (33.3%) and G190S (36.4%) increased, whereas the prevalence of L100I and K103N decreased (58.3 and 59.5%). These NNRTI-mutations became undetectable after the regimen was switched to LPV/r-based therapy (time points 7–10). Emergence of PR I54L followed by I54V was also correlated with relapse under LPV/r-based therapy. Thus, the dynamic population movements of drug-resistance mutations were successfully monitored in detail using our deep sequencing method. In addition to drug-resistance mutations, we also found a population possessing the NC/p1 cleavage-site mutation AP2V was fluctuating with the regimen switches, when we analyzed the sequences at all 11 PR cleavage sites in Gag, Pol, and Nef (Shafer and Schapiro, 2008; Fun et al., 2012). The mutation was first identified as the major population at time point 1, when SQV-based therapy was in progress. Subsequently, the mutation became a minority with EFV-based therapy, and revived as the majority with LPV/r-based therapy.

Although clinical significance was not confirmed in these two analyses of tropism testing and drug resistance mutation monitoring, our results suggest that our deep sequencing method for clinical sample analysis generates more data for understanding within-host viral co-evolutions such as tropism drifting and selection of antiretroviral resistances.

## Discussion

In this study, we have proposed a practical method to analyze viral quasispecies of the HIV-1 near-full-length genome in clinical samples using the Illumina MiSeq deep sequencing method (Supplementary Figure S6). Sequence data with low error rates are crucial for accurately analyzing minority populations and genetic diversity of HIV-1. We applied a unique error correction to minimize the effect of artificial errors and facilitate HIV-1 genome analysis using the Illumina bridge sequencing technology. Of note, Illumina bridge sequencing produces 0.52 ± 0.098% reading errors, which is significantly greater than sequencing platforms using high-fidelity polymerases (Cline et al., 1996; Palmer et al., 2005), but lower than pyrosequencing methods exhibiting high error rates at homopolymeric regions (Varghese et al., 2010; Dudley et al., 2012; Loman et al., 2012; Di Giallonardo et al., 2013; Junemann et al., 2013; Gibson et al., 2014). Our sequencing analysis of the infectious clone pNL4-3_wt_ indicated that the averaged QS-value is a reasonable guide to distinguish true and erroneous bases. One advantage of our error-correction method based on the averaged QS-value is that it removes significantly fewer nucleotides than quality-filtering error correction methods previously reported (Dudley et al., 2012; Gall et al., 2012; Pessoa et al., 2014), which increases the opportunity for detecting minority mutations.

In addition, sequencing analysis of the pNL4-3_wt_ clone suggested that reference sequence choice is critical for accurate and efficient sequence read mapping. To select an appropriate reference sequence in clinical sample analyses, we found that consensus sequence estimated from sequence reads is applicable as the reference sequence for the mapping as reported previously by others (Yang et al., 2012; Malboeuf et al., 2013; McElroy et al., 2014; Verbist et al., 2015), and that *de novo* assembly followed by iterative mapping [Schema (iv) in Supplementary Figure S1A] precisely estimates consensus sequence. During sequence analysis at PR-RT encoding regions of treatment-naïve patients' samples, *de novo* assembly followed by iterative mapping estimated the same consensus sequence as Sanger sequencing, except for one mutation in TN07 (Figure 5). By contrast, another estimation method, *de novo* assembly [Schema (ii) in Supplementary Figure S1A] inferred consensus sequences with two mutations for TN11 and another mutation for TN07.

When we performed phylogeny analyses of these estimated near-full-length consensus sequences for each clinical sample, their phylogenetic tree showed concordant subtypes to those based on their *pol* sequences by Sanger method (Supplementary Figure S7), except two samples (Non-subtype B samples #23 and #26 in Supplementary Table S1). Although these two samples were classified into CRF02_AG from the *pol* and of subtype A or G from the near-full-length sequences, this is likely due to analyzed sequence lengths and/or recombination breakpoint positions within CRF02_AG. Furthermore, phylogenetic clusters were found among samples from each drug-resistant patient. Samples from a partner pair (TN12 and TN13) diagnosed in our hospital were also phylogenetically close to each other. Taken together with high sequence identities of the Pol and Env V3 encoding regions between Sanger and our methods, these results suggest that our method may estimate consensus sequences throughout near-full-length regions accurately.

With our error-correction and mapping methods, we can obtain benefit of large genome information from the bridge sequencing. Our method enables both in-depth and semi-quantitative quasispecies analyses (Figure 5 and Supplementary Figure S5). Although we especially evaluated sequences at the Pol and Env V3 encoding regions in this study, our method would be applicable for quasispecies analyses at the other regions such at PR cleavage sites as shown in Figure 6. This is an advantage of our method that is applicable to analyze sequences throughout near-full-length genomes in depth at a run, unlike Sanger or allele specific sequencing methods. Of note, we successfully amplified genomes from low viral load samples using our designed primer sets. Therefore, when patient's viral load increases above the detection limit, our method might be helpful for early detection of drug resistant mutations. Collecting and analyzing genome date using our methods will lead to a comprehensive understanding of unknown mechanisms of resistance acquisition and treatment failure, such as the recent finding demonstrating the importance of the Env cytoplasmic tail mutation in PI resistance (Rabi et al., 2013). Moreover, our method would also be applicable to examine whether drug resistant variants are persistent as proviral DNA, although further assessment is required. Combination of *in vitro* resistance induction experiments or *in vivo* infection of HIV-1 relatives to animal models with our method would help recognize drug resistant machinery or viral evolution. However, there are at least two limitations with our analysis method. The first is due to short sequence reads. Because sequence reads in our study were up to 250-bps long, it was difficult to evaluate interferences of two or more mutations that locate more than 250-bps distant positions. Despite the limitation, our deep sequencing method could help obtain hints to know co-evolution within the genome, like mutations in PR and its cleavage sites, in combination with clonal sequencings or haplotype inference by recently proposed some bioinformatics algorithms (Beerenwinkel and Zagordi, 2011; Beerenwinkel et al., 2012; Prosperi et al., 2013; Giallonardo et al., 2014; Schirmer et al., 2014; Jayasundara et al., 2015). The second limitation is attributable to limited stocks of plasma viral RNAs. In several clinical samples, cDNA was amplified from 2.5 μL of extracted viral RNA for each of the four segments, which theoretically contained less than 100 copies of viral RNA, as the original 200 μL plasma had a viral load of < 5000 copies/mL. This limitation alerts that the results might be less heterogeneous population than in reality, although we attempted to reduce this risk by triplicate genome amplifications for deep sequencing of each sample. To address this second limitation, in addition to triplicate genome amplification, we must consider increasing several parameters, including the amount of templates, viral RNA, plasma, PCR volume, interestingly retrogressing direction of downsizing sequence technology progress in the last decade.

In conclusion, we devised a data management method and library preparation protocol to analyze quasispecies throughout the HIV-1 near-full-length genome using Illumina MiSeq bentchtop deep sequencing technology. Using deep-sequencing technology with larger genome datasets to precisely analyze minority drug-resistance mutations may improve the efficacy of antiretroviral therapy management in clinical settings.

## Accession number

The data sets analyzed in this study have been deposited in the DNA Data Bank of Japan (DDBJ) under Bioproject accession number PRJDB3502.

## Author contribution

Conceived and designed the experiments: HO, MM, KM, WS. Performed the experiments: HO, MM, KM, AH, JH, YK, YY, YI, WS. Analyzed the data: HO, MM, KM, WS. Contributed reagents/materials/analysis tools: YY, YI, WS. Wrote the paper: HO, MM, KM, WS.

## Funding

This study was supported by a Grant-in-Aid for AIDS research from the Ministry of Health, Labour, and Welfare of Japan (H25-AIDS-004) and the Research Program on HIV/AIDS from the Japan Agency for Medical Research and Development, AMED.

### Conflict of interest statement

The authors declare that the research was conducted in the absence of any commercial or financial relationships that could be construed as a potential conflict of interest.
