# Supplementary material for: Quasispecies Analyses of the HIV-1 Near-full-length Genome With Illumina MiSeq
Source: Front Microbiol. 2015 Nov 12;6:1258. doi: 10.3389/fmicb.2015.01258 (PMC4641896; doi:10.3389/fmicb.2015.01258)
Supplement: Supplementary file 1 [file Table1.PDF]

Supplementary Table S1. Clinical information for samples analyzed in this study.

	Patient #	Sample #	Sample collection date*	Regimen	Subtype	Viral load (copies/mL)	$CD4^+$ T-cell counts (cells/μL)
Treatment-naïve	1	1	Day 0		B	383000	10
	2	2	Day 0		B	722000	116
	3	3	Day 0		B	542000	23
	4	4	Day 0		B	116000	168
	5	5	Day 0		CRF01_AE	81300	59
	6	6	Day 0		CRF02_AG	24800	260
	7	7	Day 0		B	30200	35
	8	8	Day 0		B	169000	232
	9	9	Day 0		B	193000	452
	10	10	Day 0		B	96400	210
	11	11	Day 0		B	60500	163
	12	12	Day 0		B	91200	248
	13	13	Day 0		B	24000	314
	14	14	Day 0		CRF01_AE	161000	432
	15	15	Day 0		CRF01_AE	330000	3
	16	16	Day 0		CRF01_AE	230000	4
	17	17	Day 0		B	151000	76
	18	18	Day 0		B	11600	466
RAL-resistant	1	1	Day 43	FTC+TDF+RAL	B	18100	9
		2	Day 254	FTC+TDF+RAL	B	250000	130
		3	Day 317	FTC+TDF+RAL	B	4090	224
		4	Day 763	FTC+TDF+DRV/r	B	37000	181
		5	Day 807	FTC+TDF+DRV/r	B	32600	115
	2	6	Day 56	ABC+3TC+RAL	B	175000	9
		7	Day 86	ABC+3TC+RAL	B	740	64
		8	Day 106	ABC+3TC+RAL	B	24800	17
		9	Day 133	(ABC+3TC+RAL) > (FTC+TDF+DRV/r)	B	38100	10
	3	10	Day 65	FTC+TDF+RAL	B	390000	76
		11	Day 380	(FTC+TDF+RAL) > (Interrupted)	B	2700	850
		12	Day 538	Interrupted	B	98300	618
		13	Day 941	Interrupted	B	371000	134
		14	Day 1,412	(interupted) > (FTC+TDF+RAL+DRV/r)	B	416000	12
		15	Day 1,920	FTC+TDF+RAL+DRV/r	B	70800	188
	4	16	Day 861	TDF+FTC+RAL	B	88200	193
		17	Day 1,011	(TDF+FTC+RAL) > (TDF+FTC+ETR+DRV/r)	B	20600	158
	5		Day 957	ETR+RAL		65.7	375
		18	Day 1,041	ETR+RAL	B	3410	379
		19	Day 1,153	(ETR+RAL) > (ABC+3TC+LPV/r+MVC)	B	14000	186
		20	Day 1,183	ABC+3TC+LPV/r+MVC	B	187	302

Grey backgrounds show samples whose amplifications were not successful for all 4 fragments.

* Day from patient's first visit.

Supplementary Table S1. (Continued)

	Patient #	Sample #	Sample collection date*	Regimen	Subtype	Viral load (copies/mL)	$CD4^+$ T-cell counts (cells/μL)
PI-resistant	1	1	Day 768	AZT+d4T+SQV+RTV	B	5800	202
		2	Day 814	AZT+d4T+SQV+RTV	B	14400	266
		3	Day 1,118	AZT+d4T+EFV	B	500	0
		4	Day 1,194	AZT+d4T+EFV	B	2200	256
		5	Day 1,489	AZT+d4T+EFV	B	5400	185
		6	Day 1,557	AZT+d4T+EFV	B	7200	243
			Day 1,944	d4T+3TC+RTV+LPV	B	50	304
		7	Day 2,055	d4T+3TC+RTV+LPV	B	400	326
		8	Day 2,263	d4T+3TC+RTV+LPV	B	2000	218
		9	Day 2,347	d4T+3TC+RTV+LPV	B	3700	279
		10	Day 2,630	d4T+3TC+RTV+LPV	B	800	346
	2	11	Day 44	d4T+3TC+ABC	B	3500	195
		12	Day 531	ddI+d4T+LPV+RTV	B	18400	57
		13	Day 1,483	(AZT+3TC+NVP+LPV+RTV) > (TDF+FTC+NVP+T20+TPV)	B	16600	0
	3	14	Day 1,217	ddI+d4T+EFV+SQV+RTV	B	14700	400
		15	Day 1,700	ddI+d4T+EFV+SQV+RTV	B	97000	1999
		16	Day 2,098	ddI+d4T+EFV+SQV+RTV	B	40700	240
		17	Day 2,420	ddI+d4T+EFV+SQV	B	19100	137
	4	18	Day 611	d4T+3TC+SQV+NFV	B	39300	313
		19	Day 1,663	d4T+3TC+SQV+NFV	B	17600	477
		20	Day 1,922	ddI+d4T+NVP	B	19000	383
		21	Day 2,181	ddI+d4T+NVP	B	40100	371
		22	Day 2,816	ddI+TDF+FPV	B	71600	126
		23	Day 3,159	ddI+TDF+FPV	B	70600	126
Non-subtype B	1	1	Unknown		C	432	Unknown
	2	2	Unknown		C	332000	Unknown
	3	3	Day 0		C	Unknown	Unknown
	4	4	Day 0		C	15000	26
	5	5	Day 0		C	6700	411
	6	6	Day 0		C	600000	65
	7	7	Unknown		C	755000	101
	8	8	Day 0		C	254000	7
	9	9	Day 0		C	98000	238
	10	10	Day 0		C	Unknown	Unknown
	11	11	Unknown		CRF01_AE	3200	Unknown
	12	12	Unknown		CRF01_AE	1700	Unknown
	13	13	Unknown		CRF01_AE	700	470
	14	14	Unknown		CRF01_AE	2600	Unknown
	15	15	Unknown		CRF01_AE	4200	66
	16	16	Unknown		CRF01_AE	800	266
	17		Day 2,483	DRV/r+RAL+MVC	F	950	145
			Day 2,497	DRV/r+RAL+MVC	F	319	153
			Day 2,539	DRV/r+TDF+FTC+MVC	F	1240	205
		17	Day 2,869	Stribild (EVG+TDF+FTC)	F	2980	165
	18	18	Day 0		F	50000	Unknown
	19	19	Day 0		F	Unknown	Unknown
	20	20	Unknown		F	Unknown	Unknown
	21	21	Unknown		F	Unknown	Unknown
	22	22	Day 0		F	480000	409
	23	23	Day 0		CRF02_AG	210000	Unknown
	24	24	Day 0		CRF02_AG	488000	Unknown
	25	25	Day 0		CRF02_AG	1400000	Unknown
	26	26	Day 0		CRF02_AG	755000	101
	27	27	Day 0		CRF02_AG	56000	159
	28	28	Day 0		CRF02_AG	21000	186
	29	29	Day 0		CRF02_AG	360000	Unknown
	30	30	Unknown		CRF02_AG	1600	Unknown
	31	31	Day 0		CRF02_AG	50100	524

Grey backgrounds show samples whose amplifications were not successful for all 4 fragments.

* Day from patient's first visit.
