# Supplementary material for: Quasispecies Analyses of the HIV-1 Near-full-length Genome With Illumina MiSeq
Source: Front Microbiol. 2015 Nov 12;6:1258. doi: 10.3389/fmicb.2015.01258 (PMC4641896; doi:10.3389/fmicb.2015.01258)
Supplement: Supplementary file 2 [file Table2.PDF]

Supplementary Table S2. List of primer sets for genome amplification by RT-PCR (1st) and nested PCR (2nd).

			Sequences (5'->3')	Length (bp)	HXB2 loci (5'->3')
gag-rt	1st	F*	ATCTCTAGCAGTGGCGCCCGAACAG	25	625-649
	1st	R	GCTATTAAGTCTTTTGATGGGTCATA	26	3529-3504
	2nd	F	CTCTCTCGACGCAGGACTCGGCTTG	25	681-705
	2nd	R	TACTTCTGTTAGTGCTTTGGTTCC	24	3425-3402
rt-in	1st	F	ATGATAGGGGGAATTGGAGGTTT	23	2388-2410
	1st	R	CCTGTATGCAGACCCCAATATG	22	5264-5243
	2nd	F	GACCTACACCTGTCAACATAATTGG	25	2485-2509
	2nd	R	CCTAGTGGGATGTGTACTTCTGAACTTA	28	5219-5192
in-env v5	1st	F	CAGACTCACAATATGCATTAGG	22	4039-4060
	1st	R	GCCCATAGTGCTTCCTGCTGCTCCCAAGAACC	32	7786-7755
	2nd	F	CTGGCATGGGTACCAGCACACAA	23	4146-4168
	2nd	R	TATATAATTCACTTCTCCAATTGTC	25	7677-7653
env v3-nef	1st	F	GAGCCAATTCCCATACATTATTGT	24	6855-6878
	1st	R	CACTCAAGGCAAGCTTTATTGAGGC	25	9630-9606
	2nd	F	TTATTGTGCCCCAGCTGGTTTTGC	24	6872-6895
	2nd	R	GGTCTAACCAGAGAGACCCAGTACAG	26	9556-9531

* F and R denote forward and reverse primers, respectively.
