# Supplementary material for: Quasispecies Analyses of the HIV-1 Near-full-length Genome With Illumina MiSeq
Source: Front Microbiol. 2015 Nov 12;6:1258. doi: 10.3389/fmicb.2015.01258 (PMC4641896; doi:10.3389/fmicb.2015.01258)
Supplement: Supplementary file 3 [file Table3.PDF]

Supplementary Table S3. Summary of deep sequencing results for plasmid-based analyses.

(**A**) pNL4-3$_{wt}$ clone

		MiSeq read information			Mapping information	RT K65R		
Trial #	Sample #	# reads	avg. read length (bp)	avg. of QSs	Minimum coverage (*gag-nef*)	AAA>AGA (%)		codons (64-66)
						Not corrected	Corrected	Consensus
1		480346	237.1	34.5	5423	0.1	ND	AAGAAAAAA
2		783578	243.2	35.3	9227	0.0	ND	AAGAAAAAA
3		926968	243.3	35.4	11115	0.1	ND	AAGAAAAAA
4		691052	236.5	34.0	7895	0.0	0.0	AAGAAAAAA

ND: Not detected

(**B**) Mixtures of pNL4-3$_{wt}$ and pNL101-based mutant clones

		MiSeq read information			Mapping information
Trial #	Sample #	# reads	avg. read length (bp)	avg. of QSs	Minimum coverage (*gag-nef*)
1	WT-100.0-MUT-0.0	579554	245.5	32.6	5615
	WT-99.99-MUT-0.01	777080	247.3	33.6	7487
	WT-99.9-MUT-0.1	658074	247.1	33.4	6282
	WT-99.5-MUT-0.5	468172	246.0	32.0	4501
	WT-99.0-MUT-1.0	666220	247.5	32.9	6627
	WT-90.0-MUT-10.0	630686	247.1	33.5	6864
	WT-80.0-MUT-20.0	464270	246.7	33.2	5474
2	WT-100.0-MUT-0.0	417176	246.0	32.4	5106
	WT-99.99-MUT-0.01	335048	244.5	32.5	3960
	WT-99.9-MUT-0.1	325068	244.4	32.4	3865
	WT-99.5-MUT-0.5	397468	245.4	31.9	4589
	WT-99.0-MUT-1.0	320006	245.0	32.3	3974
	WT-90.0-MUT-10.0	468128	245.2	33.4	5951
	WT-80.0-MUT-20.0	381110	245.1	33.1	4773
3	WT-100.0-MUT-0.0	510436	246.3	33.2	6771
	WT-99.99-MUT-0.01	540836	246.3	33.7	7340
	WT-99.9-MUT-0.1	513744	247.2	33.6	6749
	WT-99.5-MUT-0.5	403626	245.4	32.4	5160
	WT-99.0-MUT-1.0	523900	246.9	33.0	6972
	WT-90.0-MUT-10.0	478194	247.0	33.3	6341
	WT-80.0-MUT-20.0	586932	246.7	33.7	7876
