# Supplementary material for: Quasispecies Analyses of the HIV-1 Near-full-length Genome With Illumina MiSeq
Source: Front Microbiol. 2015 Nov 12;6:1258. doi: 10.3389/fmicb.2015.01258 (PMC4641896; doi:10.3389/fmicb.2015.01258)
Supplement: Supplementary file 4 [file Table4.PDF]

Supplementary Table S4. Prevalence of detected amino acid mutations in clone mixtures after error correction.

				(a)	(b)	(c)	(d)	(e)	(f)	(g)
		pNL4-3wt		100	99.97	99.7	98.5	97	70	40
		pNL101mut		0	0.01	0.1	0.5	1	10	20
			Test	Freq(%)	Freq(%)	Freq(%)	Freq(%)	Freq(%)	Freq(%)	Freq(%)
Our error correction	RT	K103N	1	ND	0.06	0.25	0.88	1.41	12.47	21.87
			2	ND	ND	0.17	0.38	0.63	5.20	10.50
			3	0.11	ND	0.17	0.50	1.22	10.05	20.96
			Avg.	0.11	0.06	0.20	0.59	1.08	9.24	17.78
		M184V	1	ND	0.10	0.27	0.92	1.73	12.80	23.71
			2	ND	ND	0.28	1.10	1.49	11.31	23.02
			3	ND	ND	0.27	0.76	1.38	12.18	21.61
			Avg.	-	0.10	0.27	0.93	1.53	12.10	22.78
	IN	Q148H	1	ND	ND	0.48	1.45	1.83	13.52	23.18
			2	ND	ND	0.00	1.00	1.34	11.66	23.33
			3	ND	ND	0.34	0.77	1.42	10.65	20.79
			Avg.	-	-	0.27	1.07	1.53	11.94	22.43
No correction	RT	K103N	1	0.17	0.13	0.29	0.97	1.75	12.72	23.51
			2	0.26	0.31	0.34	1.23	1.51	11.25	22.83
			3	0.16	0.13	0.33	0.84	1.37	12.09	21.47
			Avg.	0.20	0.19	0.32	1.02	1.54	12.02	22.60
		M184V	1	0.01*	0.05	0.25	0.88	1.39	12.26	21.46
			2	0.09	0.15	0.16	0.37	0.61	5.12	10.32
			3	0.11	0.06	0.18	0.49	1.21	9.86	20.60
			Avg.	0.07	0.09	0.20	0.58	1.07	9.08	17.46
	IN	Q148H	1	0.36	0.22	0.50	1.52	1.83	13.30	22.68
			2	0.46	0.60	0.57	1.15	1.40	11.37	22.84
			3	0.39	0.29	0.37	0.86	1.45	10.45	20.33
			Avg.	0.40	0.37	0.48	1.18	1.56	11.71	21.95
Quality-filtering correction (QS > 20)	RT	K103N	1	0.02*	0.03*	0.17	0.60	1.50	12.27	23.18
			2	ND	ND	0.12*	0.85	1.39	10.80	22.43
			3	ND	0.01	0.13	0.58	1.17	11.50	21.51
			Avg.	0.02	0.02	0.14	0.68	1.36	11.53	22.37
		M184V	1	ND	0.01*	0.24	0.80	1.35	12.30	21.04
			2	0.01*	ND	0.08*	0.20	0.42	5.09	10.29
			3	0.04*	0.01*	0.12	0.47	1.19	9.70	20.34
			Avg.	0.03	0.01	0.15	0.49	0.99	9.03	17.22
	IN	Q148H	1	ND	0.04*	0.16	0.86	1.55	13.19	22.14
			2	0.02*	ND	0.08*	0.49	1.01	10.89	22.92
			3	ND	ND	0.10	0.43	1.21	9.77	20.16
			Avg.	0.02	0.04	0.11	0.59	1.26	11.28	21.74
Quality-filtering correction (QS > 30)	RT	K103N	1	ND	0.02*	0.16	0.61	1.49	12.26	23.10
			2	0.02	ND	0.12*	0.87	1.38	10.76	22.48
			3	0.05	ND	0.12	0.58	1.20	11.51	21.43
			Avg.	0.03	0.02	0.13	0.69	1.36	11.51	22.34
		M184V	1	ND	0.01*	0.22	0.80	1.34	12.06	20.76
			2	0.02*	ND	0.02*	0.21	0.40	4.96	10.05
			3	0.05*	ND	0.12	0.48	1.16	9.63	20.02
			Avg.	0.04	0.01	0.12	0.50	0.97	8.88	16.94
	IN	Q148H	1	ND	0.03*	0.16	0.87	1.55	13.35	22.39
			2	ND	ND	0.08*	0.47	1.01	11.05	23.13
			3	ND	ND	0.10	0.43	1.21	9.82	20.42
			Avg.	-	0.03	0.12	0.59	1.26	11.41	21.98

ND: Not detected

* The mutation was detected only in ≤5-fold coverage of sequence reads.
