# Supplementary material for: Quasispecies Analyses of the HIV-1 Near-full-length Genome With Illumina MiSeq
Source: Front Microbiol. 2015 Nov 12;6:1258. doi: 10.3389/fmicb.2015.01258 (PMC4641896; doi:10.3389/fmicb.2015.01258)
Supplement: Supplementary file 5 [file Table5.PDF]

Supplementary Table S5. PCR amplification results for clinical samples analyzed in this study.

	Patient #	Sample #	Subtype	Plasma (μL)	RNA extraction (μL)	PCR input (μL)	#Viruses for PCR (copies)	Proportion	Segment											
									gag-rt			*rt-in*			*in-env v5*			*env v3-nef*		
Treatment -naïve	1	1	B	200	50	2.5	3830	0.01	+	+	NT	+	+	NT	+	+	NT	+	+	NT
	2	2	B	200	50	2.5	7220	0.01	+	+	NT	+	+	NT	+	+	NT	+	+	NT
	3	3	B	200	50	2.5	5420	0.01	+	+	NT	+	+	NT	+	+	NT	+	+	NT
	4	4	B	200	50	2.5	1160	0.01	+	+	NT	+	+	NT	+	+	NT	+	+	NT
	5	5	CRF01_AE	200	50	2.5	813	0.01	+	+	NT	+	-	NT	+	+	NT	+	+	NT
	6	6	CRF02_AG	200	50	2.5	248	0.01	+	+	NT	+	+	NT	+	+	NT	+	-	NT
	7	7	B	200	50	2.5	302	0.01	+	-	NT	+	+	NT	+	+	NT	+	+	NT
	8	8	B	200	50	2.5	1690	0.01	+	+	NT	+	+	NT	+	+	NT	+	+	NT
	9	9	B	200	50	2.5	1930	0.01	+	+	NT	+	+	NT	+	+	NT	+	+	NT
	10	10	B	200	50	2.5	964	0.01	+	+	NT	+	+	NT	+	+	NT	+	+	NT
	11	11	B	200	50	2.5	605	0.01	+	+	NT	+	-	NT	+	-	NT	+	+	NT
	12	12	B	200	50	2.5	912	0.01	+	+	NT	+	+	NT	+	+	NT	+	+	NT
	13	13	B	200	50	2.5	240	0.01	+	+	NT	+	+	NT	+	-	NT	+	+	NT
	14	14	CRF01_AE	200	50	2.5	1610	0.01	+	+	NT	+	+	NT	+	+	NT	+	+	NT
	15	15	CRF01_AE	200	50	2.5	3300	0.01	+	+	NT	+	+	NT	+	+	NT	+	+	NT
	16	16	CRF01_AE	200	50	2.5	2300	0.01	+	+	NT	+	+	NT	+	+	NT	+	+	NT
	17	17	B	200	50	2.5	1510	0.01	+	+	NT	+	+	NT	+	+	NT	+	+	NT
	18	18	B	200	50	2.5	116	0.01	+	-	NT	+	+	NT	+	-	NT	+	-	NT
RAL-resistant	1	1	B	400	50	2.5	362	0.02	+	+	+	+	+	+	+	+	+	+	+	+
		2	B	400	50	2.5	5000	0.02	+	+	+	+	+	+	+	+	+	+	+	+
		3	B	400	50	2.5	81.8	0.02	+	+	+	+	+	+	+	+	+	+	+	+
		4	B	400	50	2.5	740	0.02	+	+	+	+	+	+	+	+	+	+	+	+
		5	B	400	50	2.5	652	0.02	+	+	+	+	+	+	+	+	+	+	+	+
	2	6	B	400	50	2.5	3500	0.02	+	+	+	+	+	+	+	+	+	+	+	+
		7	B	400	50	2.5	14.8	0.02	+	+	+	+	+	+	+	+	+	+	+	-
		8	B	400	50	2.5	496	0.02	+	+	+	+	+	+	+	+	+	+	+	+
		9	B	400	50	2.5	762	0.02	+	+	+	+	+	+	+	+	+	+	+	+
	3	10	B	400	50	2.5	7800	0.02	+	+	+	+	+	+	+	+	+	+	+	+
		11	B	400	50	2.5	54	0.02	+	+	+	+	+	+	+	+	-	+	+	+
		12	B	400	50	2.5	1966	0.02	+	+	+	+	+	+	+	+	+	+	+	+
		13	B	400	50	2.5	7420	0.02	+	+	+	+	+	+	+	+	+	+	+	+
		14	B	400	50	2.5	8320	0.02	+	+	+	+	+	+	+	+	+	+	+	-
		15	B	400	50	2.5	1416	0.02	+	+	+	+	+	+	+	+	+	+	+	+
	4	16	B	400	50	2.5	1764	0.02	+	+	+	+	+	+	+	+	+	+	+	+
		17	B	400	50	2.5	412	0.02	+	+	+	+	+	+	+	+	+	+	+	+
	5			400	50	2.5	1.3	0.02	+	-	-	-	-	-	-	-	-	-	-	-
		18	B	400	50	2.5	68.2	0.02	+	+	+	+	+	+	+	+	+	+	+	+
		19	B	400	50	2.5	280	0.02	+	+	+	+	+	+	+	+	+	+	+	+
		20	B	400	50	2.5	3.7	0.02	+	+	-	+	-	-	+	-	-	+	+	-

Grey backgrounds show samples whose amplifications were not succeeded for all 4 fragments.

Pink backgrounds highlighted succeeded amplifications.

NT: Not tested

Supplementary Table S5. (Continued)

	Patient #	Sample #	Subtype	Plasma (μL)	RNA extraction (μL)	PCR input (μL)	# Viruses for PCR (copies)	Proportion	Segment											
									gag-rt			*rt-in*			*in-env v5*			*env v3-nef*		
PI-resistant	1	1	B	400	50	2.5	116	0.02	+	+	+	+	+	+	+	+	+	+	+	+
		2	B	400	50	2.5	288	0.02	+	+	+	+	+	+	+	+	+	+	+	+
		3	B	400	50	2.5	10	0.02	+	-	-	+	+	-	+	-	-	+	+	-
		4	B	400	50	2.5	44	0.02	+	+	+	+	+	+	+	+	-	+	+	+
		5	B	400	50	2.5	108	0.02	+	+	+	+	+	+	+	+	+	+	+	+
		6	B	400	50	2.5	144	0.02	+	+	+	+	+	+	+	+	+	+	+	+
			B	400	50	2.5	1	0.02	-	-	-	-	-	-	+	-	-	-	-	-
		7	B	400	50	2.5	8	0.02	+	-	-	+	-	-	+	+	-	+	+	+
		8	B	400	50	2.5	40	0.02	+	+	+	+	+	+	+	+	+	+	+	+
		9	B	400	50	2.5	74	0.02	+	+	+	+	+	+	+	+	+	+	+	+
		10	B	400	50	2.5	16	0.02	+	+	+	+	+	-	+	-	-	+	+	-
	2	11	B	400	50	2.5	70	0.02	+	+	+	+	+	+	+	+	-	+	+	+
		12	B	400	50	2.5	368	0.02	+	+	+	+	+	+	+	+	+	+	+	+
		13	B	400	50	2.5	332	0.02	+	+	+	+	+	+	+	+	+	+	+	+
	3	14	B	400	50	2.5	294	0.02	+	+	+	+	+	+	+	+	+	+	+	+
		15	B	400	50	2.5	1940	0.02	+	+	+	+	+	+	+	+	+	+	+	+
		16	B	400	50	2.5	814	0.02	+	+	+	+	+	+	+	+	+	+	+	+
		17	B	400	50	2.5	382	0.02	+	+	+	+	+	+	+	+	+	+	+	+
	4	18	B	400	50	2.5	786	0.02	+	+	+	+	+	+	+	+	+	+	+	+
		19	B	400	50	2.5	352	0.02	+	+	+	+	+	+	+	+	+	+	+	+
		20	B	400	50	2.5	380	0.02	+	+	+	+	+	+	+	+	+	+	+	+
		21	B	400	50	2.5	802	0.02	+	+	+	+	+	+	+	+	+	+	+	+
		22	B	400	50	2.5	1432	0.02	+	+	+	+	+	+	+	+	-	+	+	+
		23	B	400	50	2.5	1412	0.02	+	+	+	+	+	+	+	+	+	+	+	+
Non-subtype B	1	1	C	400	50	2.5	NA	0.02	+	+	+	+	+	+	+	+	-	+	+	+
	2	2	C	200	50	2.5	NA	0.01	+	+	+	+	+	+	+	-	-	+	-	-
	3	3	C	400	50	2.5	NA	0.02	+	+	+	+	+	+	+	+	+	+	+	+
	4	4	C	200	50	2.5	150	0.01	+	+	+	+	+	+	+	-	-	+	-	-
	5	5	C	400	50	2.5	134	0.02	+	+	+	+	+	+	+	+	+	+	+	+
	6	6	C	200	50	2.5	6000	0.01	+	+	+	+	+	+	+	+	-	+	+	+
	7	7	C	400	50	2.5	NA	0.02	+	+	+	+	+	+	+	+	+	+	+	+
	8	8	C	400	50	2.5	5080	0.02	+	+	-	+	+	+	+	-	-	+	+	+
	9	9	C	400	50	2.5	1960	0.02	+	+	+	+	+	+	+	-	-	+	+	+
	10	10	C	400	50	2.5	NA	0.02	+	+	+	+	+	-	+	+	-	+	+	+
	11	11	CRF01_AE	200	100	2.5	16.0	0.005	+	+	+	+	+	-	+	-	-	+	+	-
	12	12	CRF01_AE	200	100	2.5	8.5	0.005	+	+	+	+	+	+	+	+	+	+	+	-
	13	13	CRF01_AE	200	100	2.5	3.5	0.005	+	+	+	+	+	-	+	-	-	+	+	-
	14	14	CRF01_AE	200	100	2.5	13.0	0.005	+	+	+	+	+	+	+	+	+	+	+	+
	15	15	CRF01_AE	200	100	2.5	21.0	0.005	+	+	+	+	+	+	+	+	+	+	+	+
	16	16	CRF01_AE	200	100	2.5	4.0	0.005	+	+	-	+	-	-	+	+	-	+	+	-
	17		F	400	50	2.5	19	0.02	+	-	-	+	+	+	-	-	-	+	+	-
			F	400	50	2.5	6.4	0.02	-	-	-	-	-	-	-	-	-	+	-	-
			F	400	50	2.5	24.8	0.02	+	+	-	+	+	+	-	-	-	+	+	+
		17	F	400	50	2.5	59.6	0.02	+	+	+	+	+	+	+	+	-	+	+	+
	18	18	F	400	50	2.5	NA	0.02	+	+	+	+	+	-	+	+	+	+	+	+
	19	19	F	400	50	2.5	NA	0.02	+	+	+	+	+	+	+	+	+	+	+	+
	20	20	F	400	50	2.5	NA	0.02	+	+	+	+	+	+	+	+	+	+	+	+
	21	21	F	400	50	2.5	NA	0.02	+	+	+	+	+	+	+	+	+	+	+	+
	22	22	F	200	50	2.5	4800	0.01	+	+	+	+	+	+	+	+	-	+	+	+
	23	23	CRF02_AG	400	50	2.5	4200	0.02	+	+	+	+	+	+	+	+	+	+	+	+
	24	24	CRF02_AG	400	50	2.5	NA	0.02	+	+	+	+	+	+	+	+	+	+	+	+
	25	25	CRF02_AG	400	50	2.5	28000	0.02	+	+	+	+	+	+	+	+	+	+	+	+
	26	26	CRF02_AG	200	50	2.5	NA	0.01	+	+	+	+	+	+	+	+	-	+	+	+
	27	27	CRF02_AG	200	50	2.5	NA	0.01	+	-	-	+	+	+	+	+	-	+	+	+
	28	28	CRF02_AG	400	50	2.5	NA	0.02	+	+	+	+	+	+	+	+	+	+	+	+
	29	29	CRF02_AG	400	50	2.5	7200	0.02	+	+	+	+	+	+	+	+	+	+	+	+
	30	30	CRF02_AG	400	50	2.5	32	0.02	+	+	-	+	+	+	+	+	-	+	+	-
	31	31	CRF02_AG	400	50	2.5	20000	0.02	+	+	+	+	+	+	+	+	+	+	+	+

Grey backgrounds show samples whose amplifications were not succeeded for all 4 fragments.

Pink backgrounds highlighted succeeded amplifications.

NA: Not available
