# Supplementary material for: Quasispecies Analyses of the HIV-1 Near-full-length Genome With Illumina MiSeq
Source: Front Microbiol. 2015 Nov 12;6:1258. doi: 10.3389/fmicb.2015.01258 (PMC4641896; doi:10.3389/fmicb.2015.01258)
Supplement: Supplementary file 6 [file Table6.PDF]

Supplementary Table S6. The number of nucleotide mismatches between primer sequences listed in Supplementary Table S3 and the Los Alamos HIV-1 subtype reference sequences.

		gag-rt				*rt-in*			
		1st*		2nd		1st		2nd	
		F†	R	F	R	F	R	F	R
Subtype B	B.FR.83.HXB2_LAI_IIIB_BRU.K03455	0	0	0	0	0	0	0	0
	B.NL.00.671_00T36.AY423387	0	1	1	2	0	0	0	0
	B.TH.90.BK132.AY173951	ND$	1	0	0	0	0	0	1
	B.US.98.1058_11.AY331295	ND	0	ND	0	0	0	0	1
Subtype C	C.BR.92.BR025_d.U52953	ND	2	1	3	2	2	0	2
	C.ET.86.ETH2220.U46016	0	0	1	3	1	2	0	1
	C.IN.95.95IN21068.AF067155	0	1	1	3	1	3	1	1
	C.ZA.04.04ZASK146.AY772699	0	2	1	4	1	1	1	2
Subtype F1	F1.BE.93.VI850.AF077336	ND	0	3	4	0	3	1	1
	F1.BR.93.93BR020_1.AF005494	ND	1	2	4	0	4	1	1
	F1.FI.93.FIN9363.AF075703	ND	1	1	5	0	2	2	2
	F1.FR.96.96FR_MP411.AJ249238	ND	0	ND	4	1	0	0	1
CRF01_AE	01_AE.AF.07.569M.GQ477441	ND	1	ND	4	1	2	0	1
	01_AE.CN.05.05GX001.GU564221	ND	1	ND	5	0	1	1	1
	01_AE.TH.90.CM240.U54771	1	1	1	4	0	1	0	2
CRF02_AG	02_AG.CM.99.pBD6_15.AY271690	0	4	3	3	0	1	0	1
	02_AG.LR.x.POC44951.AB485636	0	3	3	3	0	0	0	1
	02_AG.NG.x.IBNG.L39106	0	2	2	3	0	0	0	1

		in-env v5				*env v3-nef*			
		1st		2nd		1st		2nd	
		F	R	F	R	F	R	F	R
Subtype B	B.FR.83.HXB2_LAI_IIIB_BRU.K03455	0	0	0	0	0	0	1	0
	B.NL.00.671_00T36.AY423387	0	0	1	0	2	14	2	2
	B.TH.90.BK132.AY173951	0	0	0	1	0	0	1	0
	B.US.98.1058_11.AY331295	0	0	1	0	1	0	1	2
Subtype C	C.BR.92.BR025_d.U52953	1	1	2	0	2	ND	3	2
	C.ET.86.ETH2220.U46016	1	0	2	0	2	0	2	4
	C.IN.95.95IN21068.AF067155	2	0	2	0	2	0	2	2
	C.ZA.04.04ZASK146.AY772699	1	2	1	1	3	0	2	2
Subtype F1	F1.BE.93.VI850.AF077336	0	4	1	1	3	ND	2	1
	F1.BR.93.93BR020_1.AF005494	1	1	1	0	1	0	2	1
	F1.FI.93.FIN9363.AF075703	1	2	1	1	1	ND	2	2
	F1.FR.96.96FR_MP411.AJ249238	2	4	1	2	1	ND	2	ND
CRF01_AE	01_AE.AF.07.569M.GQ477441	0	1	1	0	1	ND	3	ND
	01_AE.CN.05.05GX001.GU564221	0	1	1	1	2	0	3	2
	01_AE.TH.90.CM240.U54771	0	1	1	0	2	0	3	2
CRF02_AG	02_AG.CM.99.pBD6_15.AY271690	1	3	1	0	0	0	0	3
	02_AG.LR.x.POC44951.AB485636	1	2	1	0	1	1	1	2
	02_AG.NG.x.IBNG.L39106	1	1	1	0	0	0	0	2

* 1st: RT-PCR, 2nd: nested-PCR

† F and R denote forward and reverse primers, respectively.

$ No sequence was determined.
