# Supplementary material for: Quasispecies Analyses of the HIV-1 Near-full-length Genome With Illumina MiSeq
Source: Front Microbiol. 2015 Nov 12;6:1258. doi: 10.3389/fmicb.2015.01258 (PMC4641896; doi:10.3389/fmicb.2015.01258)
Supplement: Supplementary file 7 [file Table7.PDF]

Supplementary Table S7. Summary of deep sequencing results for clinical samples analyzed in this study.

			MiSeq read information			Mapping information		RT K65R		
	Patient #	Sample #	# reads	avg. read length (bp)	avg. of QSs	Proportion of mapped reads (%)	Minimum coverage (*gag-nef*)	AAA>AGA (%)		Codons (64-66)
								Not corrected	Corrected	Consensus
Treatment-naïve	1	1	757636	233.2	36.0	99.7	2493	0.0	ND	AAGAAAAAA
	2	2	571534	226.1	36.1	99.7	2455	0.0	0.0	AAGAAAAAG
	3	3	782588	228.4	36.3	99.8	2973	0.0	0.0	AAGAAAAAG
	4	4	727170	228.0	36.2	99.7	3347	0.0	ND	AAGAAAAAA
	5	5	710486	237.9	35.9	97.0	3196	0.0	ND	AAGAAAAAG
	6	6	665346	235.0	36.0	99.5	3412	0.0	0.0	AAGAAAAAA
	7	7	538112	229.0	35.5	99.6	1693	0.1	0.1	AAGAAAAAA
	8	8	622126	221.4	36.3	99.7	2455	0.0	0.0	AAGAAAAAA
	9	9	603132	228.3	36.3	99.7	2155	0.0	0.0	AAGAAAAAA
	10	10	764068	222.9	36.5	99.2	2715	0.0	0.0	AAGAAAAAA
	11	11	1598524	196.0	37.0	99.8	6315	0.0	0.0	AAGAAGAAA
	12	12	1473726	197.9	36.8	99.4	6787	0.0	0.0	AAGAAAAAA
	13	13	1649546	193.6	36.8	99.8	8156	0.0	0.0	AAGAAAAAA
	14	14	1491396	194.5	37.1	99.6	9056	0.0	0.0	AAGAAAAAG
	15	15	1480630	195.5	37.1	99.5	6906	0.0	0.0	AAGAAAAAG
	16	16	1490532	199.8	37.0	99.6	8432	0.0	0.0	AAGAAAAAA
	17	17	1625828	184.5	36.4	99.6	7593	0.0	0.0	AAGAAAAAA
	18	18	1403382	188.6	36.3	98.8	5579	0.0	ND	AAGAAAAAA
RAL-resistant	1	1	447720	216.1	33.4	98.7	1619	0.1	ND	AAGAAAAAA
		2	402752	234.5	33.5	99.1	1225	0.1	ND	AAGAAAAAA
		3	427908	229.6	33.7	99.3	1603	0.1	ND	AAGAAAAAA
		4	396370	213.7	33.0	98.5	1619	0.1	ND	AAGAAAAAA
		5	351468	233.8	32.2	97.7	954	0.2	ND	AAGAAAAAA
	2	6	733690	231.6	33.5	98.9	2797	0.1	ND	AAGAAAAAA
		7	451338	235.7	33.1	99.3	2298	0.0	ND	AAGAAAAAA
		8	602638	224.2	34.4	99.6	3388	0.1	0.1	AAGAAAAAG
		9	571730	217.1	34.7	99.6	1943	0.1	0.1	AAGAAAAAG
	3	10	598506	238.1	34.2	99.8	2287	0.1	ND	AAGAAAAAA
		11	746762	235.7	35.0	99.7	3470	3.6	3.6	AAGAAAAAA
		12	791234	242.3	35.0	99.7	3452	0.1	ND	AAGAAAAAA
		13	1102536	235.4	34.5	99.5	5340	0.1	ND	AAGAAAAAA
		14	576660	233.2	33.6	99.3	2589	0.1	ND	AAGAAAAAA
		15	669770	222.5	34.6	99.5	3048	0.1	ND	AAGAAAAAA
	4	16	429736	237.0	33.8	99.6	2091	98.7	99.0	AAGAGAAAA
		17	651766	236.4	34.5	99.5	3249	99.2	100.0	AAGAGAAAA
		18	654022	234.2	34.1	99.5	3390	0.1	ND	AAGAAAAAG
		19	914632	229.1	35.1	99.4	4488	0.0	0.1	AAGAAAAAG
		20	963880	236.2	35.0	99.5	4438	0.1	0.1	AAGAAAAAG

Yellow backgrounds highlight samples whose minimum coverage throughout the *gag-nef* were <1,000-fold.

ND: Not detected

Supplementary Table S7. (Continued)

	Patient #	Sample #	MiSeq read information			Mapping information		RT K65R		
			# reads	avg. read length (bp)	avg. of QSs	Proportion of mapped reads (%)	Minimum coverage (*gag-nef*)	AAA>AGA (%)		Codons (64-66)
								Not corrected	Corrected	Consensus
PI-resistant	1	1	530872	239.6	34.6	99.3	2087	0.1	0.1	AAGAAAAAA
		2	554716	239.7	34.9	99.4	2165	0.0	ND	AAGAAAAAG
		3	504448	238.2	34.8	97.5	2095	0.1	ND	AAGAAAAAG
		4	587226	234.4	33.8	97.2	2791	0.1	ND	AAGAAAAAG
		5	354376	232.5	33.7	98.9	1430	0.1	ND	AAGAAAAAG
		6	539274	243.4	34.8	99.3	946	0.1	ND	AAGAAAAAG
		7	509040	242.1	34.5	98.3	3370	0.1	0.1	AAGAAAAAG
		8	475630	241.0	33.2	97.3	1609	0.1	ND	AAGAAAAAG
		9	389508	220.2	33.8	98.5	2337	0.1	ND	AAGAAAAAG
		10	629874	242.9	35.2	98.6	3331	0.0	0.0	AAGAAAAAG
	2	11	524824	197.1	35.5	99.5	2081	0.0	0.0	AAGAAAAAA
		12	515212	224.9	35.2	98.5	1468	0.0	ND	AAGAAAAAG
		13	490242	235.8	33.8	99.3	1520	0.1	ND	AAGAAGAAA
	3	14	262976	216.8	34.3	99.2	1196	0.1	ND	AAGAAAAAA
		15	587762	239.3	35.2	99.6	2244	0.0	ND	AAGAAAAAA
		16	561638	226.5	35.1	99.5	1903	0.0	ND	AAGAAAAAA
		17	559678	238.8	33.6	98.9	1809	0.1	ND	AAGAAAAAA
	4	18	588026	239.5	35.1	99.3	1730	0.0	ND	AAGAAAAAA
		19	643172	241.9	34.0	99.1	1972	0.1	ND	AAGAAAAAA
		20	306226	210.4	35.0	99.4	1297	0.1	0.1	AAGAAAAAG
		21	453178	225.3	35.4	99.5	1618	0.1	ND	AAGAAAAAG
		22	920840	233.8	35.8	99.7	3922	0.0	0.0	AAGAAAAAG
		23	368898	222.5	34.1	99.3	1495	0.1	0.1	AAGAAAAAG
Non-subtype B	1	1	212250	246.3	34.9	99.6	671	0.1	ND	AAAAAGAAA
	2	2	754118	247.8	35.6	99.2	4714	0.1	0.1	AAAAAGAAG
	3	3	784696	248.0	36.4	99.5	2847	0.1	ND	AAAAAGAAG
	4	4	627250	247.7	34.4	98.2	2005	0.2	ND	AAAAAGAAG
	5	5	175302	242.7	34.1	99.0	844	0.3	ND	AAAAAGAAG
	6	6	783432	246.9	35.2	99.2	3252	0.1	0.1	AAAAAGAAA
	7	7	673102	247.5	36.4	99.7	2034	0.1	0.1	AAAAAGAAG
	8	8	538112	247.2	34.1	98.3	1143	0.2	ND	AAAAAGAAG
	9	9	526140	247.5	33.8	98.2	2502	0.1	0.1	AAAAAGAAG
	10	10	775686	247.9	35.5	98.8	4578	0.1	0.1	AAAAAGAAA
	11	11	180748	237.2	34.7	98.9	710	0.2	ND	AAGAAAAAG
	12	12	222064	221.7	35.2	99.2	1075	0.2	ND	AAGAAAAAG
	13	13	239870	223.4	35.2	99.4	869	0.1	0.1	AAGAAAAAG
	14	14	295442	243.0	34.3	99.3	1828	0.1	ND	AAGAAAAAG
	15	15	306754	245.6	34.7	99.5	1180	0.1	ND	AAGAAAAAG
	16	16	192572	242.6	34.8	99.6	831	0.1	ND	AACAAAAAG
	17	17	461722	223.0	35.8	99.6	1816	0.0	ND	AAGAAAAAA
	18	18	703994	247.8	36.1	98.8	2646	0.0	ND	AAGAAAAAA
	19	19	578044	248.2	36.7	99.7	2045	0.1	0.1	AAGAAAAAA
	20	20	571460	247.6	36.2	99.7	1252	0.1	0.1	AAGAAAAAA
	21	21	590704	246.6	35.5	99.1	1990	0.1	ND	AAGAAAAAG
	22	22	637678	247.2	34.6	98.2	2477	0.1	ND	AAGAAAAAA
	23	23	247164	245.2	35.2	99.6	825	0.0	0.0	AAGAAAAAG
	24	24	488454	247.8	35.6	99.3	2581	0.0	0.0	AAGAAAAAA
	25	25	263654	246.3	35.0	99.7	1202	0.1	ND	AAGAAAAAA
	26	26	712930	247.9	35.5	99.6	3785	0.1	ND	AAGAAAAAA
	27	27	710850	247.5	35.1	99.5	3465	0.1	ND	AAGAAAAAA
	28	28	631522	247.5	34.4	97.8	2137	0.1	ND	AAGAAAAAA
	29	29	784580	247.2	35.7	99.5	2666	0.1	0.1	AAGAAAAAA
	30	30	401822	247.2	32.8	95.5	1205	0.2	ND	AAGAAAAAA
	31	31	841250	248.1	35.7	99.5	7509	0.0	0.0	AAGAAAAAA

Yellow backgrounds highlight samples whose minimum coverage throughout the *gag-nef* were <1,000-fold.

ND: Not detected
