# Supplementary material for: Quasispecies Analyses of the HIV-1 Near-full-length Genome With Illumina MiSeq
Source: Front Microbiol. 2015 Nov 12;6:1258. doi: 10.3389/fmicb.2015.01258 (PMC4641896; doi:10.3389/fmicb.2015.01258)
Supplement: Supplementary file 8 [file Table8.PDF]

Supplementary Table S8. V3 sequences identified from deep sequencing of treatment-naïve patients' samples.

Sample ID		Frequency (%)	Sequence	FPR (%)	Tropism	Direct sequencing
TN01	hap#1	82.2	CVRPNNNTRKGIHIGPGRSIYATGEIIGNIRQAHC	24	R5	Detected
	hap#2	8.6	.................................Y.	21	R5	Not detected
	hap#3	5.3	................MSA.....A...D......	44	R5	Not detected
TN02	hap#1	72.3	CTRPNNNTRKSIHLGPGRSFLATGDIIGDIRQAHC	42.8	R5	Detected
	hap#2	17.1	.................QALY..............	97	R5	Not detected
	hap#3	5.9	....................F..............	79.5	R5	Not detected
	hap#4	1.2	...........V.......................	44.8	R5	Not detected
TN03	hap#1	84.5	CTRPNNNTRRSIHLGPGSAFYATGGIIGNIRQAHC	43	R5	Detected
	hap#2	3.4	.................K..L...E..........	38	R5	Not detected
	hap#3	2.3	.................R......E..........	42.3	R5	Not detected
	hap#4	2.3	.................K......E..........	49.7	R5	Not detected
	hap#5	2.1	............R......................	8.2	X4	Not detected
	hap#6	1.4	..................V................	23.4	R5	Not detected
TN04	hap#1	76,1	CTRPNNNTRKSIHMGPGSAFYATGEIIGNIRQAHC	51.3	R5	Detected
	hap#2	17.3	............................D......	46	R5	Not detected
	hap#3	3.1	........................D..........	64.8	R5	Not detected
	hap#4	1.7	............................D..K...	28.8	R5	Not detected
TN05	hap#1	63.0	CTRPGNNTRISMTRGPGHVFYRTGDIIGDIRKAYC	1.8	X4	Detected
	hap#2	25.6	.................................H.	1.9	X4	Not detected
	hap#3	9.2	...................L...............	2.8	X4	Not detected
TN06	hap#1	61.3	CSRPNNNTRKSIRIGPGQTFYATGDIIGKIRQAYC	90.3	R5	Detected
	hap#2	20.8	........................G..........	79.7	R5	Not detected
	hap#3	6.7	........................E..........	85.3	R5	Not detected
	hap#4	5.7	..........G.H...........A...D......	97	R5	Not detected
	hap#5	2.7	........................G...N......	77.3	R5	Not detected
TN07	hap#1	50.2	CIRPNNNTRKSITVGPGRVLYTGNIIGDIRRAYC	3.4	X4	Detected
	hap#2	31.3	...................F..............	2.2	X4	Detected
	hap#3	11.6	...........V.L.....Y..R....P......	1.7	X4	Not detected
	hap#4	5.1	...........V.L.....Y..K....P......	1.9	X4	Not detected
TN08	hap#1	68.5	CTRPNNNTRKGIHLGPMGTIYATGQIIGDIRQAHC	65.2	R5	Detected
	hap#2	4.5	....S.........A.GT.FF.TGE..........	99.3	R5	Not detected
	hap#3	4.3	............................N......	72.3	R5	Not detected
	hap#4	4.3	........................E...N......	74.6	R5	Not detected
	hap#5	3.7	.I...............S......E...N......	66.6	R5	Not detected
	hap#6	3.2	.............M..........A......K...	46.8	R5	Not detected
	hap#7	1.8	....S.........A.GT.FFT..E..........	97.8	R5	Not detected
	hap#8	1.5	.................S......E...N......	72	R5	Not detected
	hap#9	1.1	..........S...R.GS.F....E..........	89.1	R5	Not detected
	hap#10	1.1	.............M........---......K...	6.92	X4	Not detected
	hap#11	1.1	..............A.GT.FF...E..........	98.9	R5	Not detected
TN09	hap#1	67.2	CTRPNNNTRRSINIGPGQALYTTNIIGDIRQAHC	98	R5	Detected
	hap#2	24.6	.........K..H.A...................	98.6	R5	Not detected
	hap#3	5.2	.........K..H.T...................	96.7	R5	Not detected
TN10	hap#1	28.6	CTRPNNNTRKGIYIGPGRRFYVRGKIIGDIRKAHC	1.1	X4	Not detected
	hap#2	21.5	............HT....M...T.E...N..Q...	6.8	X4	Detected
	hap#3	16.0	............HM...KM...T.E...N..Q...	23.9	R5	Not detected
	hap#4	11.9	.............M....M................	0.7	X4	Detected
	hap#5	3.6	..........S.HM....T...T.E...N..Q.Y.	33.9	R5	Not detected
	hap#6	3.2	.............M........T............	1.7	X4	Not detected
	hap#7	2.8	............HM....M...T.E..........	10.1	R5	Not detected
	hap#8	2.1	............HM....M...T.E...N..Q...	20.4	R5	Not detected
	hap#9	1.3	.I..........HM...KM...T.E...N..Q...	21.2	R5	Not detected
	hap#10	1.1	............HM...KM...T.E..EN..Q...	51.8	R5	Not detected
TN11	hap#1	39.1	CTRPNNNTRKSIPMGPGRTLYATGDIIGDIRRAHC	89.1	R5	Detected
	hap#2	34.7	.................K.M...............	83.3	R5	Not detected
	hap#3	21.7	.................K.................	91.6	R5	Detected
	hap#4	2.2	.................K.I...............	34.9	R5	Not detected
TN12	hap#1	46.8	CTRPGNNTRKGIHMGPGKIFYATGAITGDIRQAHC	26.2	R5	Detected
	hap#2	39.9	....S..............................	48	R5	Detected
	hap#3	3.9	.I..S..............................	43.2	R5	Not detected
	hap#4	3.1	.................R.L...............	47.6	R5	Not detected
	hap#5	1.8	.........................V.........	19.2	R5	Not detected
	hap#6	1.2	....S............RT................	76	R5	Not detected

Pink backgrounds highlight X4-tropic viral sequences.

Supplementary Table S8. (Continued)

Sample ID		Frequency (%)	Sequence	FPR (%)	Tropism	Direct sequencing
TN13	hap#1	67.1	CTRPSNNTRKGIHMGPGKTFYATGAITGDIRQAHC	80.5	R5	Detected
	hap#2	16.4	..........................I........	77.1	R5	Not detected
	hap#3	14.3	.................R.................	76	R5	Not detected
TN14	hap#1	35.8	CTRPSNNTRTSITIGPGQVFFY-RGDIIGDIRQAYC	16.6	R5	Detected
	hap#2	17.6	.........K............-.............	13.1	R5	Detected
	hap#3	16.2	.........K.........L..R.............	6.8	X4	Not detected
	hap#4	12.7	......................-.........K...	9.6	X4	Not detected
	hap#5	11.2	......................-......N......	19.4	R5	Not detected
	hap#6	1.2	...........T..........-......N..L...	6	X4	Not detected
TN15	hap#1	16.4	CIRPSSKTKMKMTMGPGKV-YYSTGEITGDIRKAHC	0.5	X4	Not detected
	hap#2	14.9	.T...NN.RKSVPI...R.-F.R..D.I....R...	9.6	X4	Not detected
	hap#3	14.4	.....NN.RTSTPI...R.-F.R..D.I........	5.7	X4	Detected
	hap#4	10.7	.....NN.RTSTPI...R.-F.R....I........	4.8	X4	Not detected
	hap#5	9.5	.T.................-F....K..........	0.2	X4	Not detected
	hap#6	8.0	.....NN.RTSTLI...R.-F.R..D.I....R...	1.7	X4	Not detected
	hap#7	7.3	.....NN.RTSTPI...R.-F.R..D.I....R...	3.2	X4	Detected
	hap#8	2.3	.....NN.RTSTPI...R.-F.K..D.I....R...	1.7	X4	Not detected
	hap#9	1.8	.T...NN.RKSVSI...R.-F.R..D.I....R...	4	X4	Not detected
	hap#10	1.6	.....NN.RKSVPI...R.-F.R..D.I....R...	8.5	X4	Not detected
	hap#11	1.4	............AM...K.-................	0.4	X4	Not detected
	hap#12	1.4	.T...NN.RTSINI...Q.LFYRPGD.I.N..E.Y.	95.5	R5	Not detected
TN16	hap#1	51.6	CTRPSNVTRKAVHTGPGRGQVFFRTGQIIGDIRKAYC	0.8	X4	Detected
	hap#2	23.3	......N..TG......Q--......E..........	13.7	R5	Detected
	hap#3	21.8	......N..TG......Q--.Y....E..........	8.3	X4	Detected
TN17	hap#1	79.2	CTRPNNNTRKGIHIGPGR-SFYTTGDIIGDIRKAYC	19.4	R5	Detected
	hap#2	5.4	..................-.............R...	7.9	X4	Not detected
	hap#3	4.4	..................W.V....K......R...	1.7	X4	Not detected
	hap#4	2.7	..................W.V....N..........	6.1	X4	Not detected
	hap#5	2.3	..................-.............R.H.	9	X4	Not detected
TN18	hap#1	98.5	CTRPNNNTRKGIHIGPGQAFYATGDIIGNIRQAHC	93.8	R5	Detected

Pink backgrounds highlight X4-tropic viral sequences.
