# Supplementary material for: Quasispecies Analyses of the HIV-1 Near-full-length Genome With Illumina MiSeq
Source: Front Microbiol. 2015 Nov 12;6:1258. doi: 10.3389/fmicb.2015.01258 (PMC4641896; doi:10.3389/fmicb.2015.01258)
Supplement: Supplementary file 9 [file Image1.PDF]

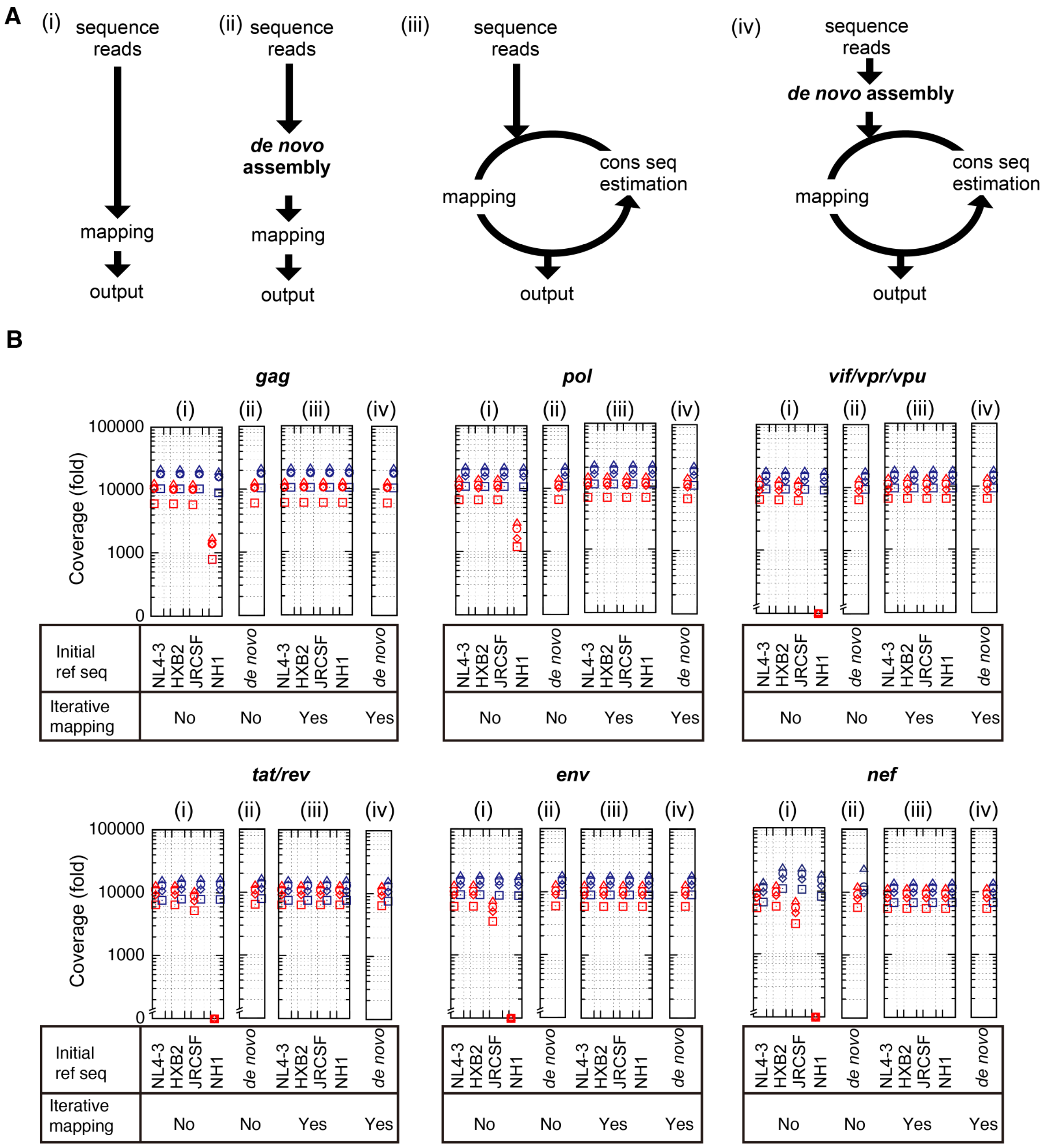

Supplementary Figure S1. Analytical biases in the sequence read mapping. (**A**) Workflow schemas of the four mapping methods. (**i**) Simple mapping on a given reference sequence. (**ii**) Mapping on a consensus sequence generated from *de novo* assembly. (**iii**) Mapping on a consensus sequence generated from iterative mapping. (**iv**) Mapping on a consensus sequence generated from *de novo* assembly followed by iterative mapping. (**B**) The red and blue symbols represent the minimum and maximum coverage in each region, respectively. The sequence read coverage from four deep sequencing runs are plotted with symbols of squares, triangles, rhombuses, and circles.
