# Supplementary material for: Quasispecies Analyses of the HIV-1 Near-full-length Genome With Illumina MiSeq
Source: Front Microbiol. 2015 Nov 12;6:1258. doi: 10.3389/fmicb.2015.01258 (PMC4641896; doi:10.3389/fmicb.2015.01258)
Supplement: Supplementary file 10 [file Image2.PDF]

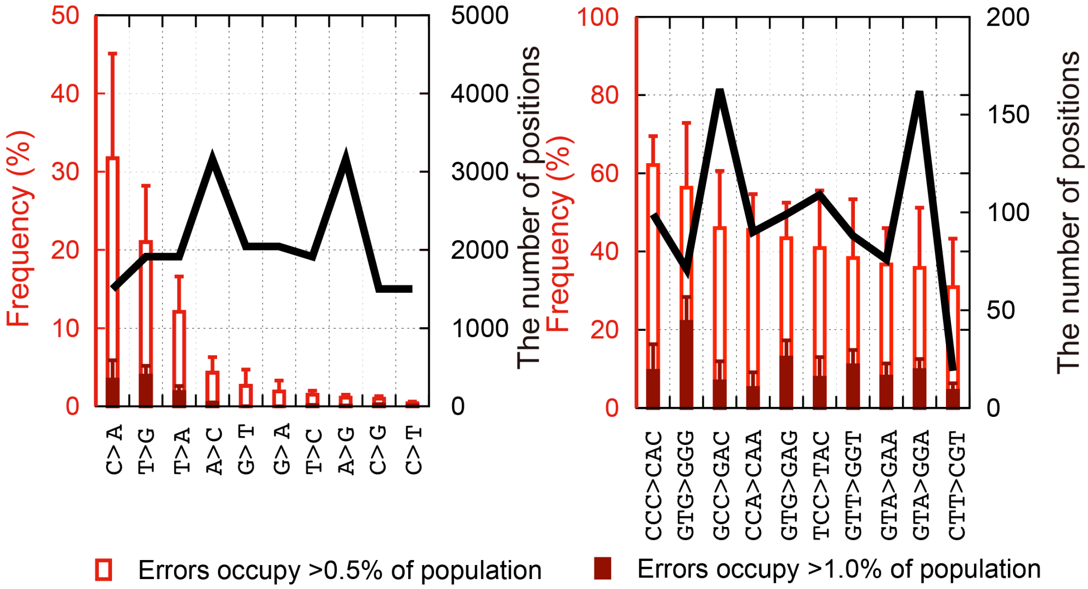

Supplementary Figure S2. Intrinsic errors of deep sequencing. Shown are the error patterns occupying >0.5% (light red open box) or >1% (dark red close box) of the population appearing in sequence reads without any error correction and the number of positions with the sequence patterns where errors frequently appeared in HIV-1 pNL4-3$_{wt}$ genome (black line). One-nucleotide sequence patterns (left) or three-nucleotide sequence patterns (right) are shown.
