# Supplementary material for: Quasispecies Analyses of the HIV-1 Near-full-length Genome With Illumina MiSeq
Source: Front Microbiol. 2015 Nov 12;6:1258. doi: 10.3389/fmicb.2015.01258 (PMC4641896; doi:10.3389/fmicb.2015.01258)
Supplement: Supplementary file 11 [file Image3.PDF]

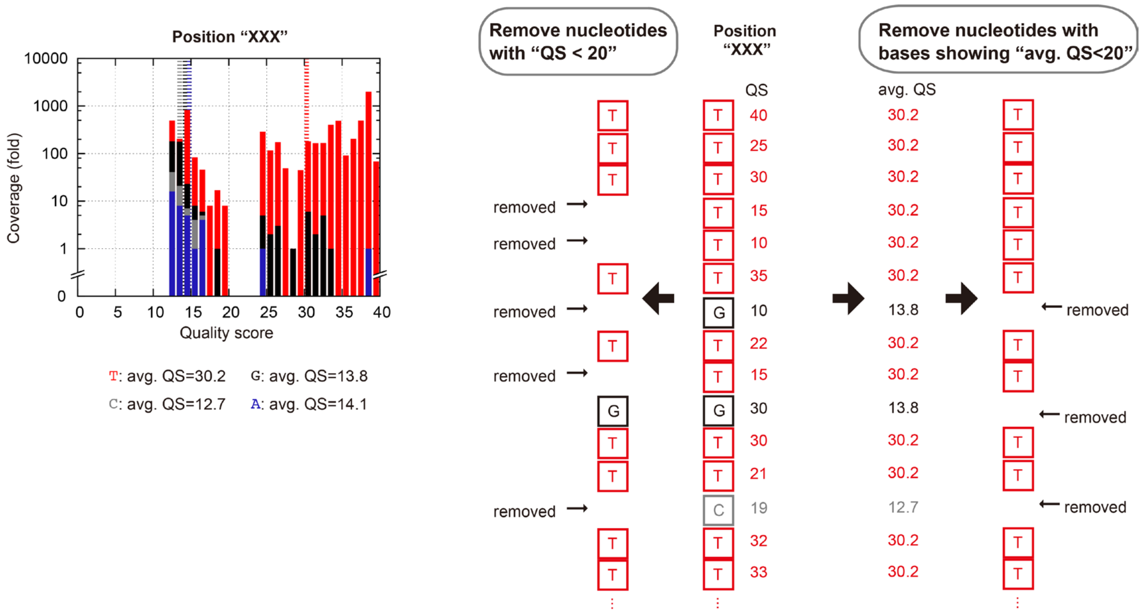

Supplementary Figure S3. An example histogram of QSs for nucleotides with each base and schema of error correction using either quality-filtering method or our method. In the left figure, red bars represent the number of nucleotides with a true base of "T", whereas black, grey, and blue bars show the number of nucleotides with erroneous bases "G", "C", or "A", respectively. The perpendicular dotted lines indicate the respective averaged QS values for "T", "G", "C", and "A" with the same colors as the bars. The right figure highlights details of the method for quality-filtering error correction by simply removing nucleotides with QSs below 20 and our method, which removes nucleotides with each base exhibiting averaged QS below 20.
