# Supplementary material for: Quasispecies Analyses of the HIV-1 Near-full-length Genome With Illumina MiSeq
Source: Front Microbiol. 2015 Nov 12;6:1258. doi: 10.3389/fmicb.2015.01258 (PMC4641896; doi:10.3389/fmicb.2015.01258)
Supplement: Supplementary file 12 [file Image4.PDF]

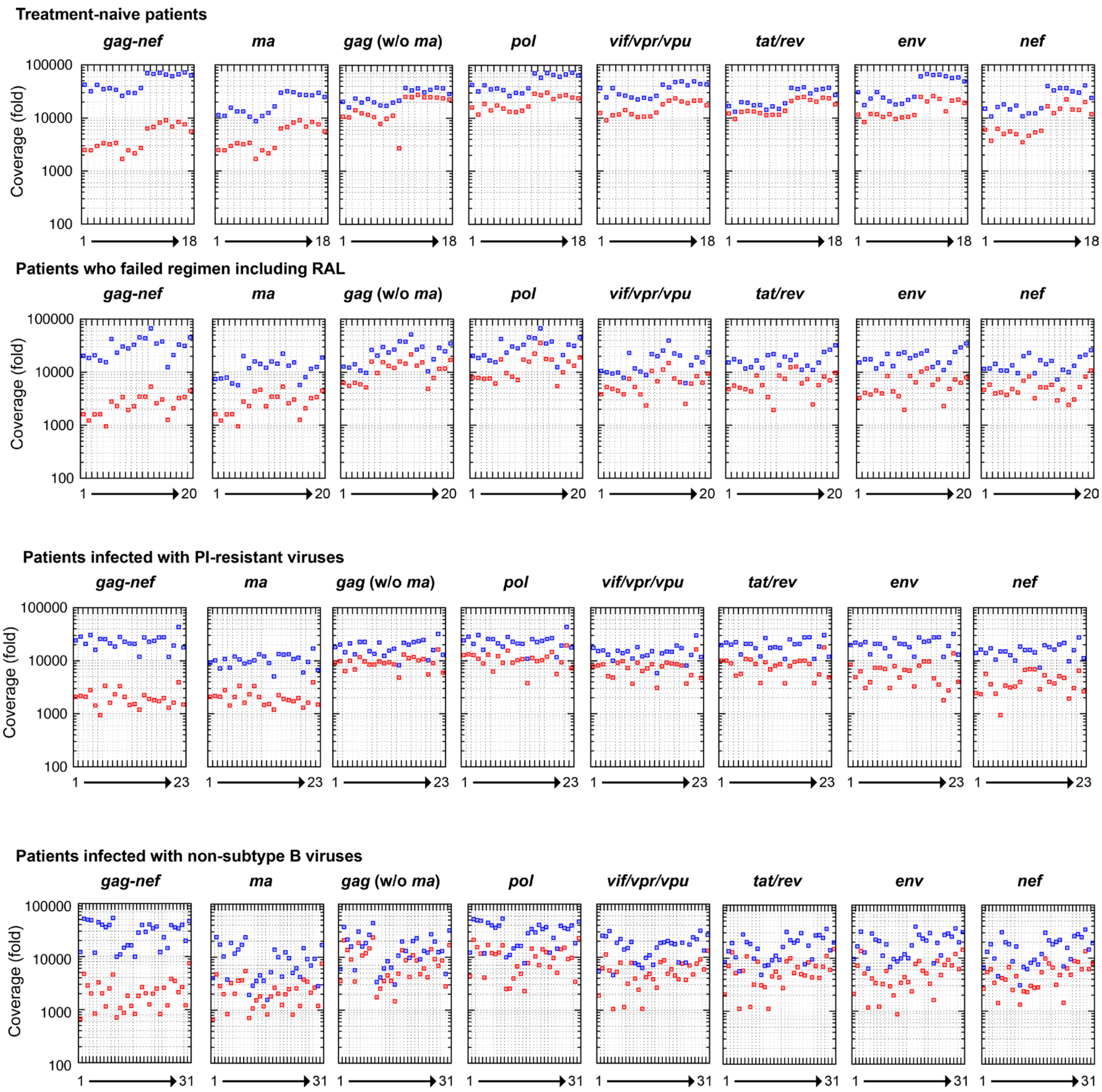

Supplementary Figure S4. Sequence read mapping onto consensus sequences for deep sequencing analysis of clinical samples. The red and blue squares in the graphs represent the minimum and maximum coverage in each region, respectively. The numbers under the graphs show samples' identification numbers listed in Supplementary Table S7.
