# Supplementary material for: Quasispecies Analyses of the HIV-1 Near-full-length Genome With Illumina MiSeq
Source: Front Microbiol. 2015 Nov 12;6:1258. doi: 10.3389/fmicb.2015.01258 (PMC4641896; doi:10.3389/fmicb.2015.01258)
Supplement: Supplementary file 13 [file Image5.PDF]

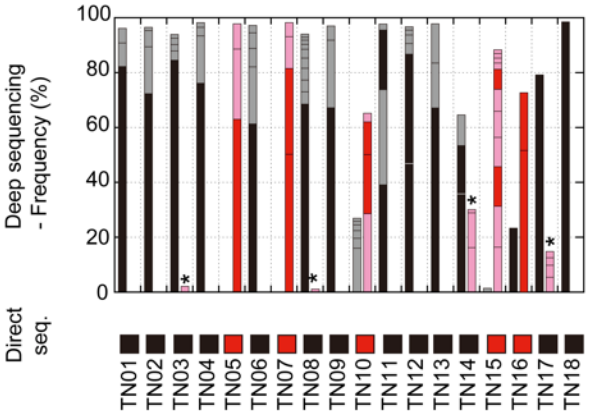

Supplementary Figure S5. Application of our deep sequencing method in genotypic tropism tests. Comparison of genotypic tropism test results between deep sequencing and Sanger sequencing. The upper graphs represent occupancies of V3 sequences calculated from the deep sequencing method. X4- or R5-tropic viral sequences detected by Sanger sequencing are highlighted with red and black bars, whereas X4- or R5-tropic viral V3 sequences unidentified by Sanger sequencing are shown with pink and grey bars. The bottom schema indicates the tropism test results from Sanger sequencing. Red and black boxes highlight the results of samples containing or lacking X4-tropic viruses, respectively. The asterisks in the upper graph highlight tropism test results discordant with Sanger sequencing results.
