# Supplementary material for: Quasispecies Analyses of the HIV-1 Near-full-length Genome With Illumina MiSeq
Source: Front Microbiol. 2015 Nov 12;6:1258. doi: 10.3389/fmicb.2015.01258 (PMC4641896; doi:10.3389/fmicb.2015.01258)
Supplement: Supplementary file 14 [file Image6.PDF]

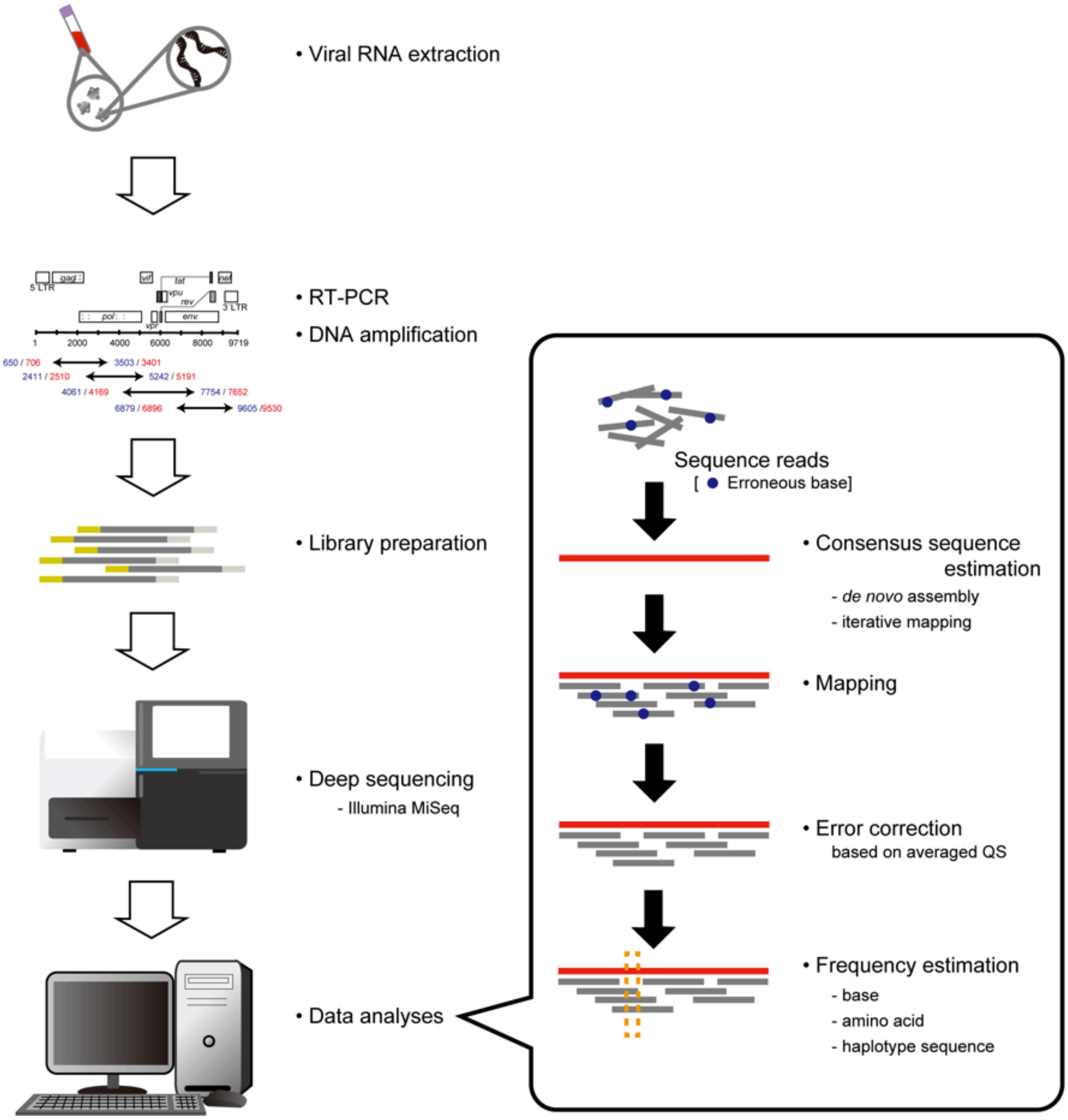

Supplementary Figure S6. Flow chart of our practical method to analyze viral quasispecies of the HIV-1 near-full-length genome in clinical samples using the Illumina MiSeq deep sequencing method. Viral RNA extracted from plasma samples was reverse-transcribed and amplified with RT-PCR and PCR reactions, with our designed primer sets listed in Supplementary Table S2. Then, library was prepared by using the amplified DNA. Next, deep sequencing was achieved with Illumina MiSeq. The obtained sequence reads were used to analyze within-host viral quasi-species, by the following four steps. (I) Consensus sequence estimation with *de novo* assembly followed by iterative mapping. (II) Mapping sequence reads onto the consensus sequence. (III) Error correction based on the averaged QSs for each base at the respective consensus sequence positions. (IV) Frequency estimation of bases, amino acids, or haplotype sequences at the respective consensus sequence regions.
