# Supplementary material for: Quasispecies Analyses of the HIV-1 Near-full-length Genome With Illumina MiSeq
Source: Front Microbiol. 2015 Nov 12;6:1258. doi: 10.3389/fmicb.2015.01258 (PMC4641896; doi:10.3389/fmicb.2015.01258)
Supplement: Supplementary file 15 [file Image7.PDF]

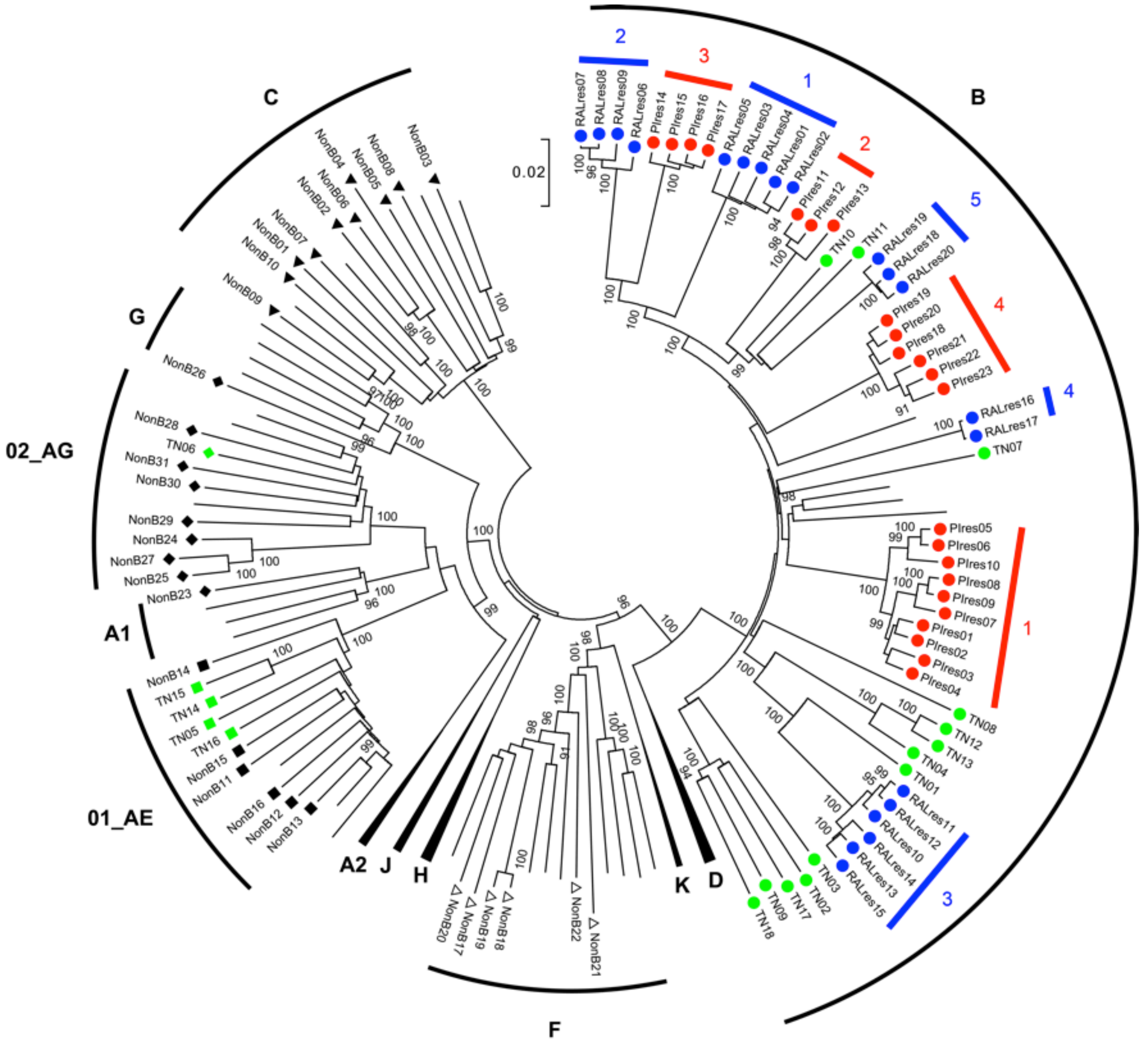

Supplementary Figure S7. Phylogeny analyses of near-full-length consensus sequences for clinical samples by deep sequencing analyses. The consensus sequences were aligned with ClustalW in MEGA6 (http://www.megasoftware.net/), along with the reference sequences (A-D, F-H, J, K, CRF01_AE, and CRF02_AG) recommended by the Los Alamos HIV sequence database (http://www.hiv.lanl.gov). Phylogeny tree was drawn using the neighbor-joining (NJ) method and the maximum composite likelihood model in MEGA6 with a bootstrap of 1,000 replicates. In the tree, bootstrap values over 90% were shown. Treatment-naïve, raltegravir (RAL)-resistant, protease inhibitor (PI)-resistant, non-subtype B patients' samples were highlighted with green, blue, red, and black signs, while samples of subtype B, subtype C, CRF01_AE, subtype F, and CRF02_AG assigned by *pol* sequences were shown with closed circles, closed triangles, closed squares, open triangles, and closed rhombuses, respectively. Branches without any signs indicate the reference sequences. Samples from same patients showing RAL-resistance or PI-resistance were further highlighted with blue and red bars and patients' identification numbers listed in Supplementary Table S1.
